# Modelling the effects of cold temperature during the reproductive stage on the yield of chickpea (*Cicer arietinum* L.)

**DOI:** 10.1007/s00484-021-02197-8

**Published:** 2021-10-05

**Authors:** Muhuddin Rajin Anwar, David J. Luckett, Yashvir S. Chauhan, Ryan H. L. Ip, Lancelot Maphosa, Marja Simpson, Annie Warren, Rosy Raman, Mark F. Richards, Georgina Pengilley, Kristy Hobson, Neroli Graham

**Affiliations:** 1 NSW Department of Primary Industries, Wagga Wagga Agricultural Institute, Pine Gully Road, Wagga Wagga, NSW 2650 Australia; 2 Graham Centre for Agricultural Innovation (an alliance between NSW Department of Primary Industries and Charles Sturt University), Wagga Wagga, NSW 2650 Australia; 3 Department of Agriculture and Fisheries (DAF), Kingaroy, QLD 4610 Australia; 4 School of Computing and Mathematics, Charles Sturt University, Wagga Wagga, NSW 2650 Australia; 5 NSW Department of Primary Industries, 1447 Forest Road, Orange, NSW 2800 Australia; 6 NSW Department of Primary Industries, 4 Marsden Park Road, Calala, NSW 2340 Australia

**Keywords:** APSIM, Australia, Flowering time, Sowing date, Field trials

## Abstract

During the reproductive stage, chilling temperatures and frost reduce the yield of chickpea and limit its adaptation. The adverse effects of chilling temperature and frost in terms of the threshold temperatures, impact of cold duration, and genotype-by-environment-by-management interactions are not well quantified. Crop growth models that predict flowering time and yield under diverse climates can identify combinations of cultivars and sowing time to reduce frost risk in target environments. The Agricultural Production Systems Simulator (APSIM-chickpea) model uses daily temperatures to model basic crop growth but does not include penalties for either frost damage or cold temperatures during flowering and podding stages. Regression analysis overcame this limitation of the model for chickpea crops grown at 95 locations in Australia using 70 years of historic data incorporating three cultivars and three sowing times (early, mid, and late). We modified model parameters to include the effect of soil water on thermal time calculations, which significantly improved the prediction of flowering time. Simulated data, and data from field experiments grown in Australia (2013 to 2019), showed robust predictions for flowering time (*n* = 29; *R*^2^ = 0.97), and grain yield (*n* = 22; *R*^2^ = 0.63–0.70). In addition, we identified threshold cold temperatures that significantly affected predicted yield, and combinations of locations, variety, and sowing time where the overlap between peak cold temperatures and peak flowering was minimal. Our results showed that frost and/or cold temperature–induced yield losses are a major limitation in some unexpected Australian locations, e.g., inland, subtropical latitudes in Queensland. Intermediate sowing maximise yield, as it avoids cold temperature, late heat, and drought stresses potentially limiting yield in early and late sowing respectively.

**Supplementary Information:**

The online version contains supplementary material available at 10.1007/s00484-021-02197-8.

## Introduction

Globally, chickpea (*Cicer arietinum* L.) production ranks second among the pulse crops after common beans (*Phaseolus vulgaris* L.). It is cultivated commercially as a cool-season crop in climates ranging from the Mediterranean to subtropical and tropical. Global chickpea production was about 17.2 Mt in 2018 from 17.8 Mha (FAOSTAT 2020). In Australia, chickpea is suited to medium rainfall (300–500 mm) areas, with slow growth during the cold winter months, accelerating in spring with warmer temperatures. Australia is the largest exporter of desi chickpeas in the world (www.pulseaus.com.au). The area under chickpeas increased to over 1 Mha in 2018 due to higher grain prices and its use as a profitable option to break disease cycles in cereal rotations. However, the average yield of chickpea in Australia is 1.1 t/ha due to diseases and abiotic stresses such as moisture, frost, and heat stress (GRDC 2011; ABARES 2020).

Chickpea is an indeterminate dicotyledonous crop, where flowering is spread over a relatively long period, with leaf and branch production continuing while flowering and pod-filling proceed (Unkovich et al. 2010; Peake et al. 2020). Chickpea plants exposed to unfavourable conditions during the reproductive phase can abort flowers, young pods, or developing seeds and resume flowering when conditions improve (Clarke and Siddique 2004). Unfavourable conditions include frosts (< 0 °C screen temperature) and/or less-well-defined periods of cold temperatures (referred to as chilling) (Croser et al. 2003; Yadav et al. 2010). Chickpea is unlike other pulses and exhibits particular sensitivity to cold temperature, and improving this trait is a major target for breeding programs around the world (Abbo et al. 2003; Croser et al. 2003; Yadav et al. 2010; Berger et al. 2012). Chickpeas can set pods when the minimum temperature is < 5 °C as long as the maximum day temperature is > 20 °C (Srinivasan et al. 1998; Singh et al. 2021). Some authors refer to mean day temperatures < 15 °C, not minimum temperature, as sufficient to cause cold damage (Siddique and Sedgley 1986; Berger et al. 2004), whereas other controlled environment studies refer to minimum temperatures below 15 °C as sufficient to cause cold damage (Croser et al. 2003; Clarke and Siddique 2004). Cold temperature sensitivity is less likely to manifest in controlled environment studies, but becomes important in field trials, particularly in the northern grain-growing regions of Australia comprising parts of northern New South Wales and Queensland (lower latitudes) due to warm winter days. If chickpeas experience daytime temperatures below 20 °C and/or night temperatures below 10 °C, then floral abortion may still occur, along with poor pod-filling. Therefore, a combination of these environmental conditions will lead to reduced grain yield if the plant cannot compensate for fewer seeds by increasing seed weight (Srinivasan et al. 1998; Clarke and Siddique 2004; Nayyar et al. 2005; Kumar et al. 2010). These temperature thresholds are problematic not just in Australia but also in other chickpea growing regions, including northern India (Clarke and Siddique 2004; Nayyar et al. 2005).

The reproductive phase is sensitive to abiotic stresses, including cold and is crucial in determining the adaptation of various crops, including pulses, to diverse environmental conditions (Lake et al. 2021). In addition to extreme temperatures, the soil water status of the crop is known to affect flowering time (Richards et al. 2020) significantly, and this parameter is now included in the latest version of APSIM for chickpeas (Classic version 7.10). In the past, multi-location experiments were used to fine-tune crop management practices such as, time of sowing to minimise risk of abiotic stresses and screening for crop adaptation (Turner 2004; Crespo-Herrera et al. 2018; Gerard et al. 2020). Multi-location experiments can be time-consuming and expensive and can lead to errors such as non-representative sampling of locations and years and are usually influenced by genotype-by-environment interactions (Chapman et al. 2000a, b; Chapman et al. 2002). The field testing of genotype (G), environment (E), and management (M) interactions (G × E × M) experimentally is usually severely limited by the number of factor combinations that can realistically be evaluated.

In contrast, cropping system productivity under variable G × E × M scenarios can be evaluated using in silico crop modelling tools, such as APSIM (Hall and Richards 2013; Grassini et al. 2015). Also, crop modelling may help identify gaps in existing knowledge and highlight topics worthy of further scientific investigation. Therefore, the main objectives of this study were to (i) validate the predictive ability of the current APSIM-chickpea model for flowering time and grain yield using field-experiment data in a broader range of target production environments than previously attempted; (ii) characterise and understand chickpea flowering time and its interaction with frost using a yield-gap analysis approach; and (iii) estimate the impacts of various cold temperatures on the reproductive stage of chickpea and on potential grain yield.

## Materials and methods

### Field experiments

Chickpea crops were established in 29 field experiments in 10 locations (Table 1) across five States: South Australia (SA), Victoria (VIC), New South Wales (NSW), and Queensland (QLD) using a commercial seeder, following the cultural practices as per National Variety Trials protocol (https://www.nvtonline.com.au/nvt-protocols/) for nutrition and control of diseases, pests and weeds. Since the experiments are diverse, there was some variation in row spacing, sowing depth, targeted plant densities, hand-harvested area, sample processing, and agronomic details (see Table 1). Observations on flowering were made when 50% of the plants in a plot had at least one open flower. The yield was determined at plant maturity and converted into kg/ha. Daily meteorological data (including maximum and minimum air temperature (°C), rainfall (mm), and solar radiation (MJ/m2)) for these locations were obtained from the Scientific Information for Land Owners website (SILO) (https://legacy.longpaddock.qld.gov.au/silo/about.html; Jeffrey et al. (2001)). Additionally, climatic variables at some of the experimental sites were directly monitored in the field separately (Table S1) from early sown and main season chickpea trials across seven locations. These data replaced the climate data downloaded from SILO. The details of the soil properties at the experimental sites are given in Table S8.

**Table 1:** Metadata for 29 published and ongoing chickpea field experiments (NSW DPI and GRDC) used to calibrate and validate the APSIM-chickpea module for cultivar PBA HatTrick. Location, soil type (Isbell 2016), plant available water holding capacity (PAWC in mm), date of sowing, row spacing (RS) and plant population (PP) are listed below. SA, South Australia; QLD,Queensland; NSW, New South Wales; VIC, Victoria; NSW DPI and GRDC, Grains Agronomy & Pathology Partnership (GAPP), between the NSW Department of Primary Industries (DPI) and the Grains Research and Development Corporation (GRDC) project (BLG111)

Location, State	Soil type	PAWC	Sowing date	Agronomic details	Reference
Roseworthy, SA	Sodosol (Calcic Luvisol)	126	7 Jun. 2013 7 Jun. 2013 15 Jul. 2014	Irrigated and dryland, RS = 24 cm; PP = 55 plants/m^2^	Lake and Sadras (2017)
Billa Billa, Qld	Vertosol	183	27 May 2015 24 May 2016 29 May 2017	Dryland, RS = 100 cm; PP = 33 plants/m^2^	Chauhan et al. (2019)
Roma, Qld	Vertosol	119	19 May 2016 30 May 2017	Dryland, RS = 100 cm; PP = 33 plants/m^2^	Chauhan et al. (2019)
Wagga Wagga, NSW	Kandosol	110	24 Apr. 2014 12 May 2014 18 May 2015 17 May 2016 2 Jun. 2016 26 May 2017 18 May 2018 15 May 2019	Dryland and partly irrigated, RS = 50 cm; PP = 50 plants/m^2^	Richards et al. (2020); NSW DPI and GRDC
Yenda, NSW	Sandy Loam	165	1 May 2016 20 May 2016	Dryland, RS = 50 cm; PP = 50 plants/m^2^	GRDC
Trangie, NSW	Red chromosol	141	18 Apr. 2018 16 May 2018 30 Apr. 2019 15 May 2019	Dryland Dryland Pre and post sowing irrigation, RS = 40 cm; PP = 35 plants/m^2^	Richards et al. (2020); NSW DPI and GRDC
Tamworth, NSW	Vertosol	245	18 Apr. 2018 24 May 2016 23 May 2017 12 Jun. 2018	Dryland and partly irrigated, RS = 40 cm; PP = 35 plants/m^2^	NSW DPI and GRDC
Leeton, NSW	Brown chromosol	293	15 May 2019	Pre-sowing irrigation, RS = 50 cm; PP = 50 plants/m^2^	Richards et al. (2020); NSW DPI and GRDC
Breeza, NSW	Vertosol	255	15 May 2019	Pre-sowing irrigation, RS = 40 cm; PP = 35 plants/m^2^	NSW DPI and GRDC
Horsham, VIC	Grey cracking clay	248	16 May 2019	Dryland, RR = 35 cm; PP = 30 plants/m^2^	NSW DPI and GRDC

### APSIM-chickpea model evaluation

The Agricultural Production Systems Simulator (APSIM) Classic version 7.10 (www.apsim.info/), comprising the APSIM-chickpea module (Robertson et al. 2002), was configured to simulate the trials (Table 1) reported in this study. Detailed descriptions of APSIM are provided by Holzworth et al. (2014). The APSIM model (Holzworth et al. 2014) does not account for the effects of pests and diseases, pollen sterility, and flower/pod abortions due to extreme weather events, and cannot simulate grain production from secondary flushes of flowers (e.g., from plant compensatory growth after a damaging event such as frost or chilling temperatures. We calibrated the APSIM-chickpea module for the PBA HatTrick cultivar using all the field data (Table 1). The thermal time from emergence to the end of the juvenile phase was modified to 690°Cd, and other modifications to the parameters are listed in Table S2 (Supplementary material). Plant available water capacity (PAWC) was calibrated as the difference between the field capacity water and 15 bar lower limit for each soil.

To predict the flowering, the current chickpea module of APSIM uses a thermal time approach only. However, the thermal time approach deficiently predicted flowering time, as we observed a discrepancy of about ± 10 to ± 31 days between observed versus simulated flowering times (Anwar et al. 2019; Chauhan et al. 2019). Furthermore, the involvement of soil water in modulating flowering of chickpea has recently been demonstrated (Chauhan et al. 2019). We therefore used a modified version of the APSIM to simulate chickpea flowering time by including soil moisture interactions, in addition to temperature and photoperiod (Chauhan et al. 2019) using the following equation:

1 $$\mathrm{TTm}=\mathrm{TT}\times(1.65-\mathrm{FASW})(\mathrm{when}\;\mathrm{FASW}\geq0.65\;\mathrm{and}\;\mathrm{the}\;\mathrm{chickpea}\;\mathrm{stage}\geq3)$$

In this equation, TTm is the modified thermal time, TT is the thermal time as computed by the APSIM model without soil water modification, and FASW is fractional available soil water. FASW is equal to 1 when soil water is close to the field capacity and permanent wilting occurs when it reaches 0. According to this equation, the thermal time was only modified when FASW was > 0.65 and the emergence (growth stage 3) had already been reached. This approach is different to the approach of Soltani et al. (2006) where thermal time was modified only when chickpea plants are acutely water-stressed. In our study, modification of TT was maximum when FASW was near the field capacity. FASW was computed using the following equation:

2 $$\mathrm{FASW}(n)=\Sigma(\mathrm{sw}\_\mathrm{dept}(n)-\mathrm{ll}15\_\mathrm{dept}(n))/\Sigma(\mathrm{dul}\_\mathrm{dept}(n)-\mathrm{ll}15\_\mathrm{dept}(n))$$

where sw_dept is soil water at the start of the day, ll15_dept is soil water at 1.5 MPa soil water potential, dul_dept is the soil water at the drained upper limit (i.e., field capacity), and *n* is the number of layers in the 60 cm soil surface used in soil parameterisation (Holzworth et al. 2014). In addition to TTm, we assessed the impact of frost on yield because it causes significant yield loss (Maqbool et al. 2010). In chickpea, each post-flowering frost event is estimated to cause about 5% yield loss (Chauhan et al. 2019). Frost is defined as the minimum daily temperature in the Stevenson Screen at 1.5 m aboveground level ≤ 0 °C (Chauhan et al. 2019).

The performance of APSIM-chickpea was evaluated using three measures of “goodness-of-fit”, calculated between the observed and simulated values (flowering time and grain yield in this case). These are the following:

1. the root mean square error (RMSD) (Piñeiro et al. 2008), calculated as follows:
  3 $$\mathrm{RMSD}=\sqrt{\frac{1}{N-1}{\sum }_{i=1}^{N}{\left({S}_{i}-{M}_{i}\right)}^{2}}$$
  where *S*_*i*_ is the simulated value, *M*_*i*_ is the measured value and *N* is the number of measurements across the 29 sites and seasons (Table 1). The value of RMSD closer to zero connotes a better model simulation.
2. Willmott’s index of agreement (*d*) (Willmott 1982; Loague and Green 1991) was determined by the following:
  4 $$d=1-\frac{{\sum }_{i=1}^{N}{\left({S}_{i}-{M}_{i}\right)}^{2}}{{\sum }_{i=1}^{N}{\left(\left|{S}_{i}-\overline{M}\right|+\left|{M}_{i}-\overline{M}\right|\right)}^{2}}$$
  where $\overline{M}$ is the average of *M*_*i*_ and best results are where *d* approaches unity.
3. The coefficient of determination (*R*^2^), the association between the measured and predicted values, was also evaluated.

### Simulation scenario analysis

**Fig. 1:**
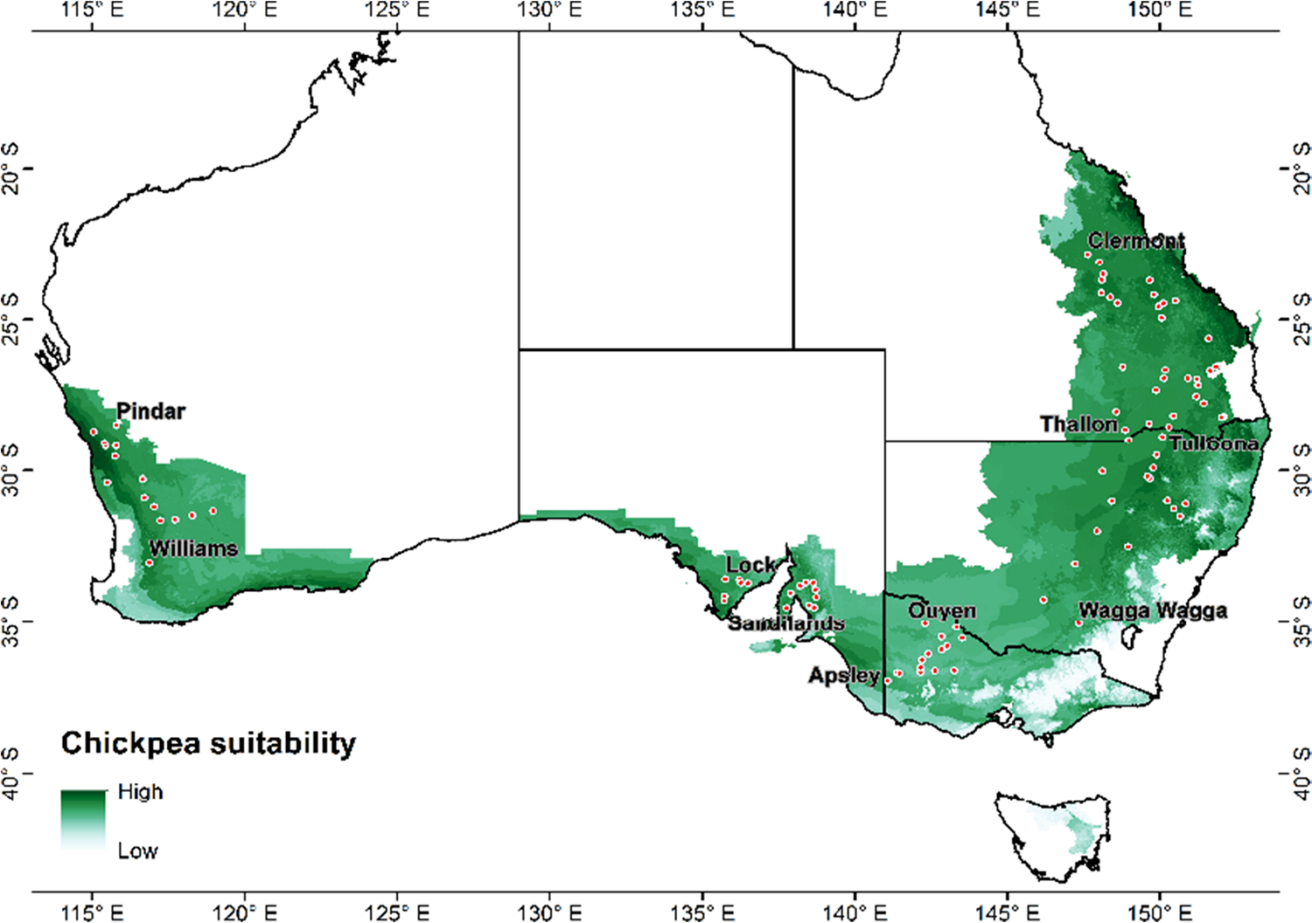
Growing areas of Australia where land suitability analysis (Table S5) showed potential for Chickpea growing (depicted in green) based on long-term climatology, soil pH and topography (Saaty 1980; Chen et al. 2010; NCI 2020). The dots show the 95 locations used in the APSIM-chickpea simulation study reported here (Table S3)

We developed a factorial simulation setup in APSIM to simulate the chickpea crop cycle, flowering time, and yield for 95 locations grown with 33 plants/m^2^ at a row spacing of 50 cm for 70 years (1950–2019). Peak flowering time was calculated as the mid-point between start and end of flowering. The factorial design consisted of 95 locations, three sowing dates (early (10–30 April), mid (1 May–21 May), and late (22 May–11 June)), and three Desi cultivars, PBA HatTrick, PBA Boundary and PBA Seamer. Two cultivars were classified as mid maturity (PBA HatTrick and PBA Seamer), whereas PBA Boundary as mid-late. The cultivar parameters used for PBA Boundary and PBA Seamer were described in Chauhan et al. (2019). These 95 locations included both National Varietal Trial sites (www.nvtonline.com.au) situated within developed commercial production areas and potential areas for chickpea cultivation based upon a detailed land suitability analysis (LSA) (Fig. 1). The details of how the LSA was conducted are given in the Supplementary Information (Table S6). Sites for simulation could not be chosen to cover the entire LSA-designated area (Fig. 1) as all the soil characteristics required for the APSIM model were not available at all locations.

Details of the selected 95 sites around Australia (Table S3 (See Supplementary Information)) include the coordinates of sites, description of soil types with water holding capacities, and the three sowing times chosen to match the usual practice of local commercial sowing based on the GRDC winter crop sowing guide (https://grdc.com.au/). Table S3 also includes summary information about rainfall, temperature, and frost incidence.

Simulations were carried out with 20 kg of N/ha applied at sowing as urea, following farmers’ practice and surface organic matter and N were reset at sowing. For a given location the model sowed the crop on the first available opportunity, i.e., when 7-day cumulative rainfall was ≥ 25 mm and the plant available soil water was > 80 mm. If these criteria are not met, sowing was assumed to occur on the last day of the sowing window which led to crops failing at that location (less than < 1% of all simulations tested), due to lack of establishment. At the start of the summer season (1^st^ November), the fractional available soil water was reset to 50% in the model to mimic the low available soil water left after a winter fallow, assuming weeds were constantly controlled using herbicides. From this time onward, until the start of the next sowing window, soils could be equilibrated with pre-season rainfall before sowing. Location-specific soils for all locations were chosen from the APSOIL database (Holzworth et al. 2014).

### Cold temperature analysis

Cold temperatures at critical reproductive stages impact chickpea yield. We wanted to estimate the association between cold temperatures and yield; therefore, we fitted multiple linear regression models (Draper and Smith 1981) across different sowing dates and cultivars (3 cultivars × 3 sowing dates) for all locations (Table S3). The response variable was the simulated frost-affected chickpea yield ($YF$) (using a 5% loss per frost event; Chauhan et al. 2019; Chauhan and Ryan 2020)) in 70 years from 1950 to 2019. The explanatory variables were the total rainfall amount ($R$) during the reproductive period (*RP*), and the percentage of days (*P*) during *RP* where the minimum temperature is below *T*_*C*_. This regression approach of assessing chilling temperature thresholds is only one of several possibilities but we believe it is simple, straightforward, and easy to test. We are aiming to detect the threshold temperatures for each site/cultivar combination across all the simulation years and we might expect these values to vary, possibly significantly. Ideally, the APSIM model should be fully parameterised for chilling and frost damage but that is not currently available.

The modelling procedure consisted of three steps:

- Step 1: Choice of possible T_C_ values
  We examined the chickpea literature which suggested that mean daily temperatures ˂15 °C during the reproductive phase can inhibit pod set, cause pod abortion, and result in subsequent yield losses (Croser et al. 2003; Berger et al. 2004). However, we defined the range of tested *T*_*C*_ values from 0.0 to 19.0 °C (inclusive) because we wanted to ensure that any sensitivity to temperature above 15 °C was captured in the analysis. Therefore, *P* was calculated as follows:
  $$P=\frac{\mathrm{Days}\;\mathrm{during}\;RP\;\mathrm{where}\;\mathrm{minimum}\;\mathrm{temperature}\leq T_c}{\mathrm{Total}\;\mathrm{number}\;\mathrm{of}\;\mathrm{days}\;\mathrm{during}\;RP}\times100\%.$$
  In this definition $P$ must be between 0 and 1 (or 0% to 100%). A regression model was then fitted according to Eq. 5.
  5 $$Y{F}_{t}={\beta }_{0}+{\beta }_{1}{P}_{t}+{\beta }_{2}{R}_{t}+{e}_{t},$$
  where $t=1950, 1951, \dots ..., 2019$ and ${e}_{t}$ is the random error term distributed normal with a mean of zero and a unit variance. The estimates and the *p*-values of ${\beta }_{1}$ were recorded.
- Step 2: Criteria-based selection of T_C_
  The cold temperature threshold, *T*_*C*_, was selected through grid search using a step of 0.1 °C within the interval between 0.0 and 19.0 °C (inclusive). At each potential value of *T*_*C*_, model specified in Eq. 5 was fitted. To avoid having multiple *T*_*C*_ values, only the one(s) which produced the lowest *p*-value of *β*_1_ in step 1 were retained. In case the same *p*-value was produced from multiple *T*_*C*_ values, the maximum of these was chosen. Two criteria were applied. Firstly, the *p*-value of *β*_1_ had to be less than 0.05. Secondly, *T*_*C*_ had to be between the first quartile and the third quartile of the minimum temperatures within the *RP* period. This prevented values for *P* being too close to 0 or 1, ensuring a wide range for *P*. With these criteria in place, it is possible that for some locations and some years there is no *T*_*C*_ detection.
- Step 3: Quantification the effects of cold temperature
  Using the selected *T*_*C*_ based on step 2, the multiple regression model in Eq. 5 was then fitted again. The estimate of ${\beta }_{1}$ can be interpreted as the change in $YF$ when $P$ is increased by 1%, assuming $R$ remains the same. If the estimate of ${\beta }_{1}$ is negative, a yield loss is expected whenever the minimum temperature falls below *T*_*C*_. The larger the absolute value of the estimated ${\beta }_{1}$, the larger the impact.

### Software

Aside from ASPIM Classic version 7.10 (as parameterised and modified as described in the text), all other data manipulation and analysis was conducted using R software (R Core Group, 2019) running under the RStudio Integrated Development Environment (RStudio 2019). Within R, a number of add-on packages were extensively used (see Supplementary References). The latest available version of R was used as the work progressed (from 3.5.2 in 2018 through to 4.1.0 in 2021), as were the latest package versions available from CRAN (Comprehensive R Archive Network, https://cran.r-project.org/mirrors.html). Some data storage was performed in Microsoft Excel spreadsheets.

## Results

### Model performance

**Fig. 2:**
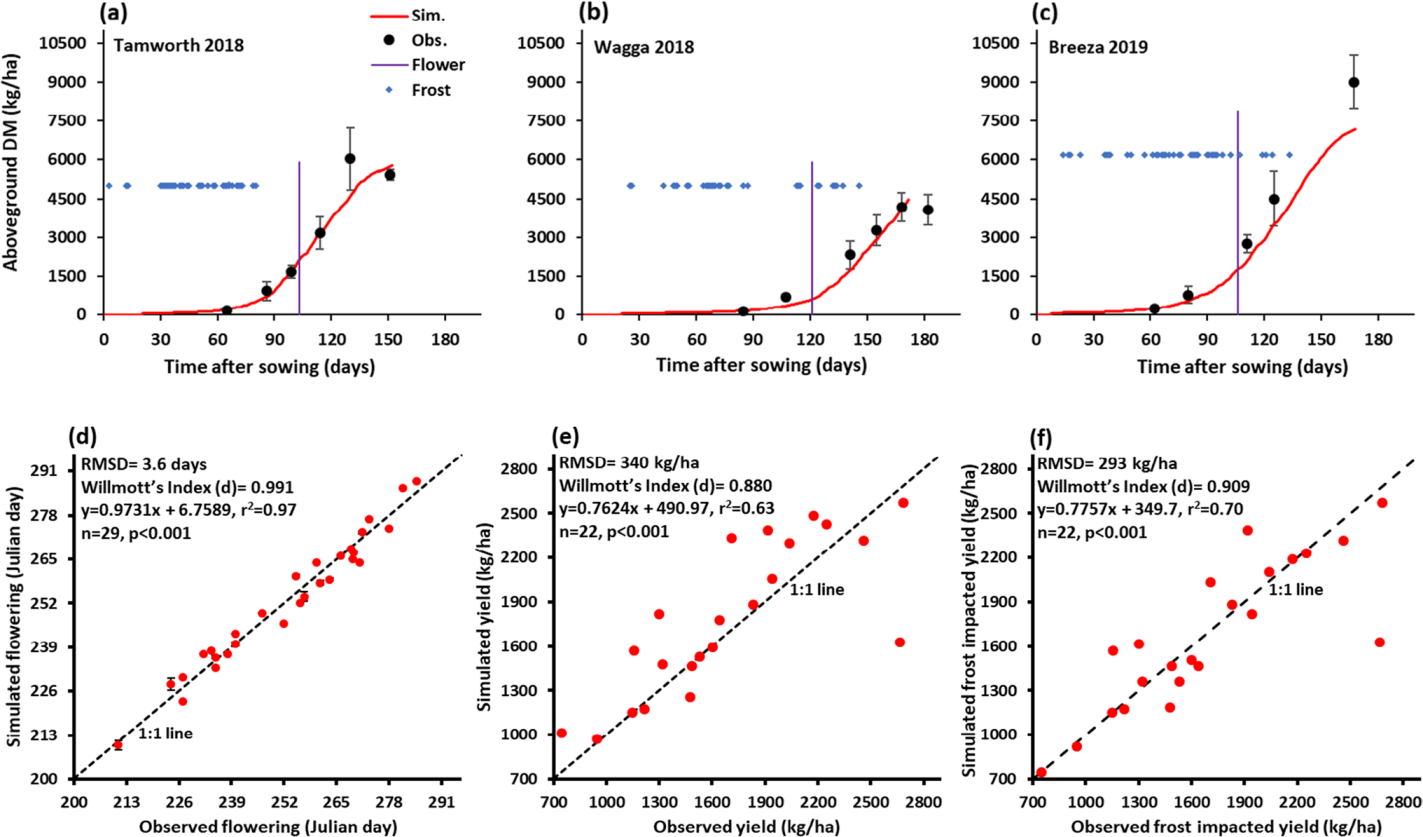
Evaluation of APSIM-chickpea for PBA HatTrick showing observed vs. simulated **a**, **b**, and **c** aboveground biomass (DM), **d** flowering time, and **e** and **f** is yield at physiological maturity using experimental data from Table 1. The dashed diagonal line is the 1:1 line and insets are the values of RMSD, *d* and linear regression details. In Figs. 2a, b, and c symbols with error bar of observed data and red line are simulated, vertical purple line is the observed flowering time and blue dots are frost events in three experimental sites. A total of 29 experiments were utilised for flowering time whereas only 22 were available for yield

Daily simulated aboveground biomass data was based upon the site-specific soils, temperature, and rainfall data recorded in Tamworth, Wagga Wagga, and Breeza (2018 and 2019) at the field experimental locations (Tables 1 and S1). The accumulation of simulated aboveground biomass (Fig. 2a–c) up to the post- flowering phase generally followed the patterns of observation across the three locations. At Tamworth in the 2018 growing season, there was no frost event during the post-flowering phase (Fig. 2a), whereas there were many post-flowering frost events in Wagga Wagga in 2018 and Breeza in 2019 (Fig. 2b–c).

The model-predicted flowering time versus the measured values from 29 field experiments (Table 1) covering a wide range of environmental conditions were similar. The APSIM-chickpea module was able to explain 97% of the observed variability in flowering time with a RMSD of 3.6 days (Fig. 2d). The Willmott’s index of agreement (*d*) value of the model-predicted flowering time versus the measured values was 0.99 (Fig. 2d) and indicated a good performance of the model in terms of predicting chickpea flowering time. The observed and simulated chickpea grain yield are illustrated in Figs. 2e and 2f along with 1:1 line. The APSIM-chickpea module was able to explain 63% (*R*^2^ = 0.63) of the variance in grain yield with a RMSD of 340 kg/ha. The fitted line diverged from the1:1 line indicating slight underestimation of grain yield (slope of the regression line = 0.76). The Willmott’s index of agreement (*d*) value for predicted chickpea grain yield (Fig. 2e) was 0.88, demonstrating the model’s reasonable predictive capacity. However, the prediction improved (*R*^2^ = 0.70, *d* = 0.91, and RMSD = 293 kg/ha) when the impact of frost was taken into account (Fig. 2f).

The observed yields ranged from about 700 to 2700 kg/ha and the model adequately predicted over this range (Fig. 2e–f). Overall, the performance parameters indicated a good agreement between measured and simulated values by APSIM-chickpea model. This provided confidence to simulate biomass accumulation, flowering time, and grain yield over a wide range of environmental conditions and suggested that this model could be applied across new potential growing areas in the following analysis.

### Modelling occurrence of flowering

**Fig. 3:**
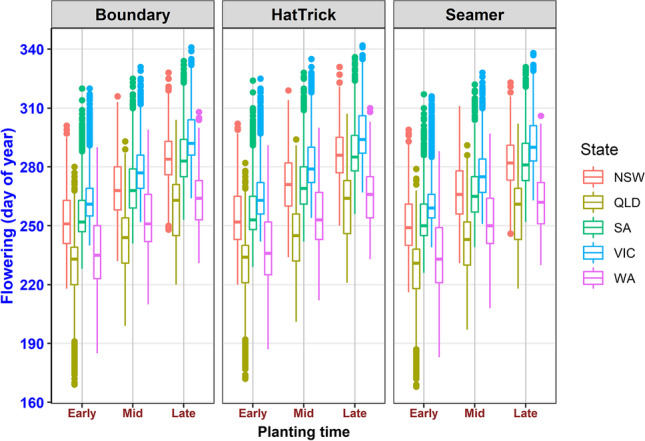
Distribution (1950–2019) of flowering time (Julian days) across five States comprising 95 locations (Table S3) and the values were group by cultivars and sowing dates. The horizontal line in each box-plot is the median value, the lower edge of a box is the 25^th^ percentile and the upper edge, 75^th^. The whiskers reach to 1.5 times the interquartile range (between the 25^th^ and 75^th^ percentiles) or to the most extreme observed value, whichever is smallest; dots below or above the whiskers represent individual values beyond this range. NSW, New South Wales; QLD, Queensland; SA, South Australia; VIC, Victoria; WA, Western Australia

Flowering time was significantly (*p* < 0.001) influenced by cultivar, sowing date, State (comprising 95 locations), PAWC (plant available water holding capacity), and yearly mean in-crop rainfall (Table S4). Figure 3 shows the median of the flowering time grouped by State and the detailed distribution of flowering time across 95 locations can be found in supplementary Figure S1. The flowering time ranged from 241 day of the year for early sown PBA Seamer to 276 for later sown PBA HatTrick averaged across all locations. In general, flowering time is influenced by temperature, photoperiod, and soil water interactions. Within a location, flowering time is affected in the same way as later sowing experience higher or lower temperature and in-season rain prior to flowering (building soil water storage) or its loss due to evaporation (where it does not rain much as in the subtropical environment.

There were differences in flowering time due to sowing date, with early sowing resulting in earlier flowering for all the three cultivars. Comparing the five States, flowering was largely early in Queensland and Western Australia compared to the other three States (Fig. 3). The average flowing time for PBA Boundary ranged from 239 in Queensland to 279 in Victoria, for PBA HatTrick, it ranged from 241 in Queensland to 281 in Victoria and for PBA Seamer, it ranged from 238 in Queensland to 276 in Victoria. For the two-way interactions, there was cultivar-by-State and sowing date-by-State interactions, but no cultivar-by-sowing date interactions were observed (Table S4). The flowering time was predicted to be delayed by about 0.16 days and advanced by about 0.02 days for each 1 mm increase in growing season rainfall and PAWC, respectively. Additionally, there was huge variability in flowering time across the 95 locations. For example, Morawa (Western Australia) had variable growing season rainfall and low minimum temperature with high PAWC (Table S3). However, the dispersion in the distribution of flowering time was larger compared to Biloela (Queensland) which had less variable growing season rainfall, higher minimum temperature with lower PAWC (Fig. S1).

### Risk of frost during reproductive phase

**Fig. 4:**
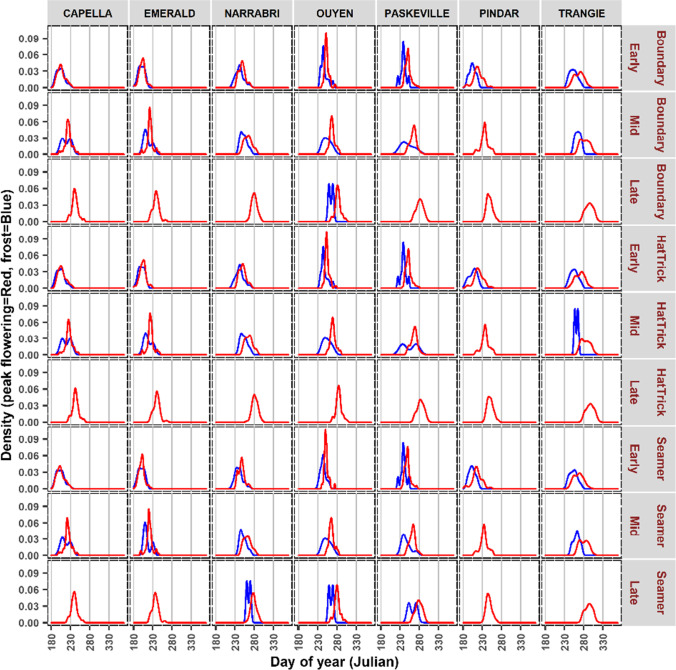
Example of a density estimates of frost events (blue line) and peak flowering time (red line) for 70 years (1950–2019). The plots were group by cultivars and sowing dates across seven locations, the same figures for rest of locations are in supplementary (Fig. S2)

The vulnerability to frost damage during peak flowering is shown as the degree of overlap of the two density curves, as a snapshot of seven locations (Fig. 4), with data for the 95 locations provided in the supplementary material (Fig. S2). Across the 95 locations, the peak flowering ranged from 200 Julian day in early sowing at Duaringa (Queensland) to 318 Julian day in late sowing at St Arnaud (Victoria). In some locations, there were no frosts (absence of a blue line), while in others, the red and blue lines are well separated indicating a lower risk of frost damage. However, for other locations, the red and blue lines closely follow each other showing maximum frost risk during the reproductive stage. For instance, when PBA HatTrick was sown later at these seven locations, it did not encounter frost incidents, while the later sown PBA Boundary was at risk of frost at Ouyen (Victoria) only (Fig. 4). Additionally, especially at Pindar (Western Australia), all three cultivars did not encounter frost incidents when mid sown, whereas at Ouyen, all cultivars except the later sown PBA HatTrick were at risk of exposure to frost damage.

### Variability of water-limited yield potential

**Fig. 5:**
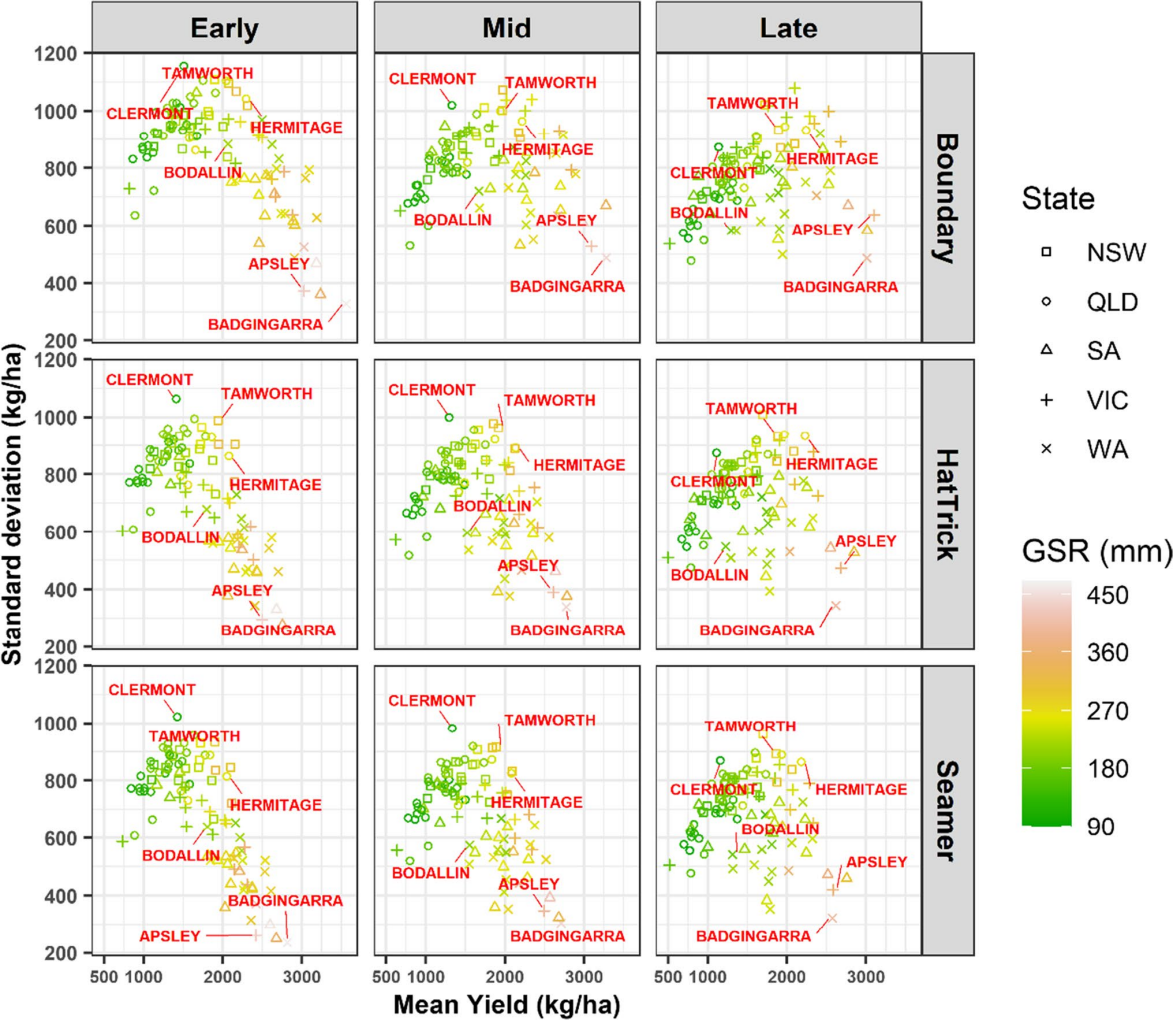
Variability of simulated chickpea yield (1950–2019) across five States comprising 95 locations (Table S3). Yield values were group by growing season rainfall, cultivars and sowing dates. As an example, six locations names are highlighted, and the details are shown in the supplementary (Fig. S3). NSW, New South Wales; QLD, Queensland; SA, South Australia; VIC, Victoria; WA, Western Australia; GSR, growing season rainfall

Chickpea yield varied (Figs. 5 and S3) according to the temperature, radiation, rainfall patterns, and soil type of each environment (Table S3) across 95 locations. Figure 5 shows the mean of the annual yield within the 70-year simulation, grouped by State that comprises 95 locations and its interaction with growing season rainfall. As an example, when cultivar PBA Boundary was planted early, the highest mean yield (3556 kg/ha; less variability compared to other locations, standard deviation = 328 kg/ha) was at Badgingarra (Western Australia), where the growing season rainfall is more than 455 mm (Fig. 5). This was followed by Apsley in Victoria (2580 kg/ha; standard deviation = 420 kg/ha), where the growing season rainfall was about 367 mm, when cultivar PBA Seamer was planted late. However, in Bodallin (Western Australia), which received 182-mm growing season rainfall and lower soil PAWC (101 mm), cultivar PBA HatTrick had an average yield of 1223 kg/ha when sown late. Across all cultivars and sowing time, Tamworth (New South Wales) and Hermitage (Queensland) were some of the mid yielding locations (1925 kg/ha and 2168 kg/ha, respectively). In contrast, lower growing season rainfall (about 102 mm) and the lowest yielding location with high annual variability was Clermont in Queensland (about 1333 kg/ha; standard deviation ≥ 998–1154 kg/ha). The detailed distribution of chickpea yield across 95 locations can be found in supplementary Figure S3.

Additionally, the yearly yield was statistically fitted on cultivar, sowing date, locations, soil type (plant available water holding capacity (PAWC in mm)), and yearly mean growing season rain using a linear model. Second-order interactions between cultivar, sowing date, and locations were included in the model. We found the analysis of variance had statistically significant effects for all variables and interactions (Table S5). Also, yield was moderated by PAWC and sowing date. However, the influence of PAWC was fairly small, especially across locations, indicating that growing season rainfall had the strongest influence on yield. The final yield was predicted to increase by 2.80 kg/ha and 5.79 kg/ha for each 1 mm increase in PAWC and growing season rainfall, respectively. Cultivar PBA Boundary provided the highest yield, in general, compared to PBA HatTrick and PBA Seamer. Later, sowing was found to be the least advantageous across all States and cultivars. Early sowing was found to work the best for cultivar PBA Boundary but the differences between sowing period were less obvious for other cultivars.

### Variability of cold temperature index

**Fig. 6:**
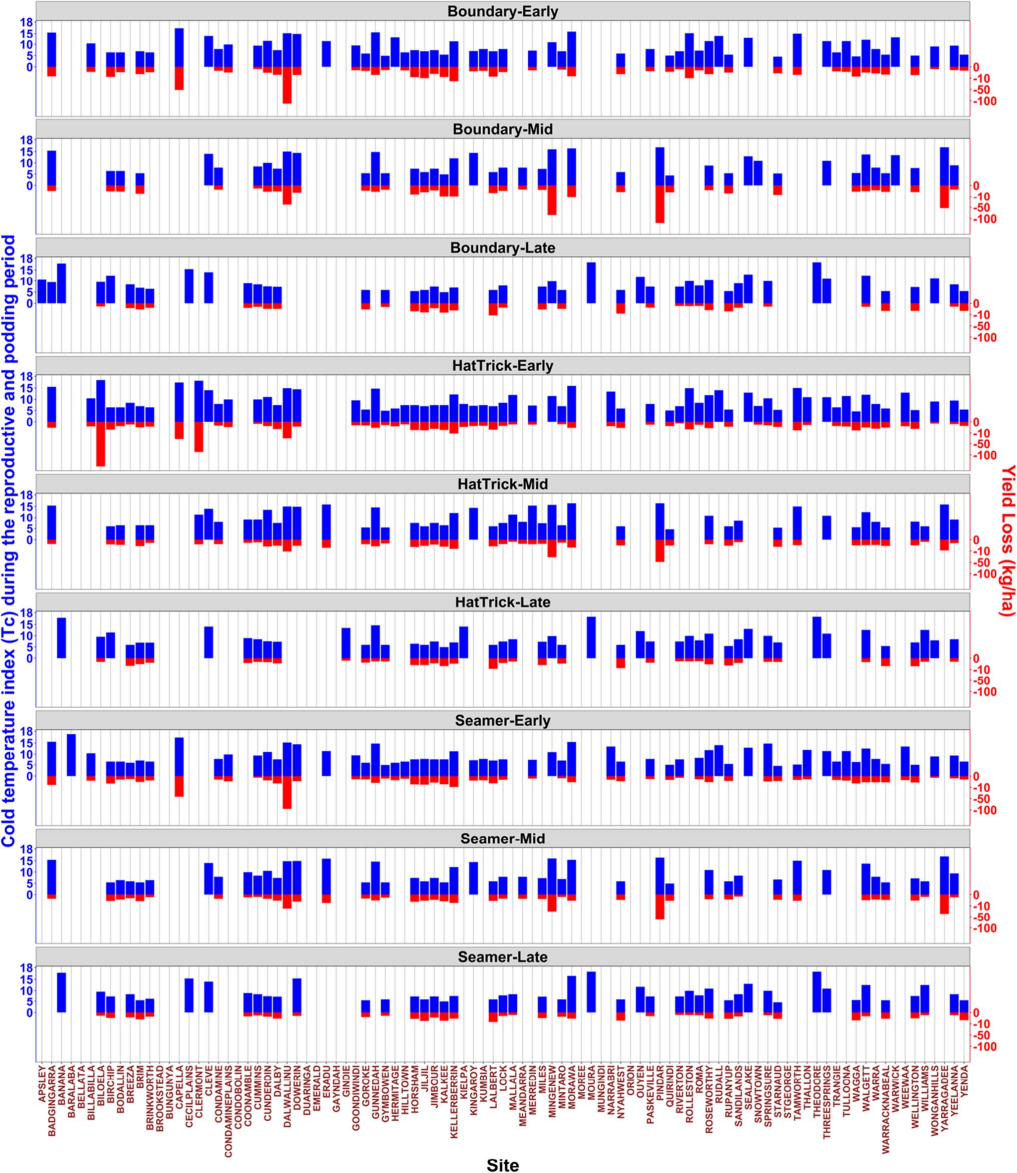
Detected (*p* < 0.05) cold temperature (*T*_*C*_) index (blue bars) during the reproductive and podding period and the corresponding yield loss (red bars; if any) by cultivars and sowing date scenarios across 95 locations (Table S3)

Figure 6 summarises the detection of cold temperatures (*T*_*C*_) across the 95 locations (by cultivar and sowing date). There were some locations (cultivar × sowing date) where *T*_*C*_ was not detected under our approach. In general, the frequency of *T*_*C*_ detection declined with later sowing dates (Fig. 6). Specifically, *T*_*C*_ was detected at locations in Cunderdin (Western Australia), Roseworthy (South Australia), Horsham (Victoria), Walgett (New South Wales), and Dalby (Queensland) as an example, when the sowing dates were early, mid and late, respectively. The detected *T*_*C*_ ranged from 4.4 to 18.9 °C (9.4 °C on average). In general, the cold temperatures were higher in Northern and Eastern areas. Also, the cold temperature threshold appeared to be higher in inland areas compared to coastal areas (Fig. S4). In the detection of *T*_*C*_, there appeared to be an interaction with soil type, rainfall, minimum temperature, and incidence of post-flowering frosty days. As an example, at Eradu (Western Australia), the soil was a loamy duplex (PAWC = 96 mm), had higher growing season rainfall (308 mm), and very low incidence of frost (May to August frost = 0.1 days yearly mean (Table S3)). We found the detected *T*_C_ was about 15.9 °C for mid sown cultivar PBA HatTrick. In contrast, at St Arnaud (Victoria), where the soil was a brown Sodosol (PAWC = 202 mm), with high incidence of frost (May to August frost = 14.9 days yearly mean), we found the detected *T*_*C*_ was 4.5 °C when PBA Seamer cultivar was sown later.

The percentage of days (*P*) between flowering to pod-fill where the minimum temperature was below *T*_*C*_ were statistically significant (*p* < 0.05) across 95 locations, were the *T*_*C*_ was detected. The effect of *P* on the yield loss ranged from − 12.3 to − 492 kg/ha across the locations where a *T*_*C*_ value was detected (Figs. 6 and S4). In general, the effect of *P* on the yield loss was greater in Eastern areas than Western areas, with the most extreme estimated effect recorded in Billa Billa (Queensland) for early sown cultivar PBA HatTrick. Additionally, Table S7 shows the correlation between *T*_*C*_ and a set of variables including latitude and longitude, PAWC, annual average minimum temperature (T) during the reproductive and podding period (RP), annual average frost days during RP, annual average growing season rainfall, annual average April to October rainfall, annual average mean T during April to October, annual average minimum T during April to October, annual average day length (hours) during April to October, and yield (kg/ha), with magnitudes greater than 0.3 are being highlighted. On an overall basis, *T*_*C*_ was more related to latitude, longitude, minimum T, and day length during April to October. Generally, both temperature and day length are closely related to latitude and longitude and were key factors in determining a region’s climate. Thus, we can argue in a way that *T*_*C*_ is spatially variable. We suspect that variabilities in temperature and day length probably reflect changes in latitude and longitude (hence the locations), especially latitude (latitude and day length had a correlation of 0.99 while latitude and temperature had a correlation of roughly 0.80).

We fitted a linear regression model for *T*_*C*_ on latitude and longitude. The fitted equation is as follows:

$${T}_{C}=42.823+0.579\times \mathrm{Latitude}-0.105\times \mathrm{Longitude}$$

Both the coefficients for latitude and longitude were statistically significant (*p* < 0.05). The *T*_*C*_ was higher towards the North (i.e., increase in latitude) and the West (i.e., decrease in longitude). Figure S5 shows a scatterplot and the pattern is very clear for latitude but less clear for longitude, which agrees with the correlations. We considered cultivars and sowing dates separately, and *T*_*C*_ was not significantly different across either cultivar (*F* = 0.382, *p* = 0.537) or sowing dates (*F* = 0.034, *p* = 0.854).

## Discussion

The use of crop models in conjunction with field experiments can improve the understanding of crop yield limitations, identification of genotypes better adapted to their environment, identify gaps in model functions, and improve current forecasting methods to better account for genotype-by-environment interactions and extreme events (Chauhan et al. 2017; Lake et al. 2021). Prior to our study, the APSIM-chickpea model was calibrated and validated for the historical chickpea cultivar (cv. amethyst) grown in the northern chickpea growing region of Australia (Robertson et al. 2002) and chickpea commercial cultivars, such as PBA Boundary, PBA HatTrick, PBA Seamer, and Tyson (Chauhan et al. 2017, 2019) based on a limited range of environments conducted in the northern region. In this study, we further finetuned the genetic coefficient of cultivar PBA HatTrick (Table S2) using 29 field experiments across diverse locations (Table 1). Our re-calibration showed good agreement between simulated and measured aboveground biomass accumulation, flowering time, and grain yield (Fig. 2). For example, we obtained *R*^2^ and RMSD values between simulated and observed flowering time were 0.97 and 3.6 days, respectively. Additionally, the coefficient of determination (*R*^2^) and RMSD improved further when the impact of frost events was incorporated into the model (*R*^2^ = 0.70, RMSD = 293 kg/ha) and the observed yields ranged from 784 to 2683 kg/ha; our model adequately predicted this range. Moreover, our coefficient of determination is much higher than 0.6 obtained in another study (Kaloki et al. 2019), and our RMSD values are less compared to Robertson et al. (2002) and Kaloki et al. (2019). This also affirms the improvements in predictions achieved in this study from the re-parameterised APSIM model. The differences between the values obtained in this study and those reported in the literature can also be explained by the different groups of cultivars but also by differences in the experimental conditions.

The current version of the chickpea module of APSIM, uses temperature and photoperiod to predict the flowering time. However, these two factors inadequately predicted flowering time; with a discrepancy of about ± 10 to ± 31 days between observed versus simulated flowering time (Anwar et al. 2019; Chauhan et al. 2019). To improve the prediction of flowering time, here we used temperature, photoperiod, and soil moisture interactions in a modified APSIM-chickpea module as reported by Chauhan et al. (2019) and Chauhan and Ryan (2020).

Despite the three cultivars being of similar maturity classification (mid for PBA Boundary, and mid-late for PBA Seamer, and PBA HatTrick), there were consistent differences in their time to flowering across different sowing times. Similar maturity classification might be due to their related pedigree; PBA HatTrick, and PBA Boundary have Jimbour as a parent while PBA HatTrick is a parent of PBA Seamer. PBA Seamer flowered earlier than PBA Boundary who was earlier than PBA HatTrick regardless of sowing time. Large *G* × *E* interactions were reported in chickpea and other crops (Berger et al. 2004; Parent et al. 2017). We observed this interaction in the form of cultivar-by-State interaction. Early or late flowering did not offer yield advantages compared to ‘intermediate’ as PBA Boundary generally provided the highest yield, when compared to the later flowering PBA HatTrick and the early flowering PBA Seamer. This may indicate that early flowering crops encounter suboptimal temperatures and frost stress while late flowering crops would encounter moisture and heat stress, which limits yield potential. Days to flowering was previously shown to be negatively correlated with yield especially at high temperatures (Devasirvatham et al. 2015). Risk of temperature impact can be minimised, by adjusting sowing time to match flowering window to minimise the risk of adverse environmental conditions (Whish et al. 2007; Maqbool et al. 2010). Additionally, short photoperiods increase thermal time requirement of the crop to flower. Because high soil water makes crop perceive lower daily thermal time, it takes longer to achieve certain increased day degree target when there is considerable soil water (due to high soil water storage or rains prior to flowering) compared to a drought situation. Lower ambient temperatures magnify this effect further by influencing the evapotranspiration demand. So, with same amount of soil water, the crop will take much longer to flower if it was growing in a cooler environment, e.g., in southern States compared to in the Central Queensland where daily ambient temperatures are higher (Singh et al. 2021).

For the three cultivars, early sowing resulted in plants flowering earlier in the year (Julian date). Sowing date was shown to affect phenological development and duration of chickpea growth phases (Richards et al. 2020). It is commonly recognised that early sowings (e.g., in autumn) increase the crop’s vegetative duration due to low temperatures and shorter days experienced in the period leading to winter. This was largely the case in our study as the risk of frost exposure decreased with late sowing (Fig. 4). However, the indeterminate nature of chickpea would still allow chickpea to yield satisfactory after stressful events such as frost if subsequent conditions are favourable. In addition, we found late sowing resulted in yield penalties across all States and cultivars (Fig. S3), a finding commonly observed. Early sowing was found to be advantageous for PBA Boundary yield but the differences between sowing time were less obvious for the other two cultivars. Additionally, we found in our ongoing field trials that sowing early can decrease yield in some situations. Sowing early in the recommended window increases the risk of vegetative frost in some (more prostrate) cultivars. Early sowing increased winter biomass which significantly increased disease risk in the southern areas. We also confirmed that the increased water use from sowing early impacts on grain yield in low rainfall or short seasons with low starting soil water (Sadras and McDonald 2012; Chauhan and Ryan 2020).

Comparing the locations in the five States, flowering was largely early in Queensland and Western Australia compared to the other three States. The large diversity of locations (Table S3) resulted in some locations encountering different levels/degrees of frost exposure, and this facilitated understanding of optimum sowing time (early, mid, or late) for any of the three cultivars at the 95 locations studied here. As an example, PBA HatTrick and PBA Boundary would be suitable for late sowing as they avoided potential exposure to frost incidents (except at location Ouyen (Victoria) for PBA Boundary). Also, all the three cultivars can be sown at mid sowing window at Pindar (Western Australia) location.

The critical period for yield determination is centred around flowering (Lake and Sadras 2014), and therefore environmental conditions experienced during the reproductive phase are major determinants of final grain yield. Abiotic stresses such as frost, heat, and drought limit yield potential through inducing flower abortion, pod drop, or failure of grain development (Maphosa et al. 2020). Ultimately, this results in fewer pods or high number of unfilled pods, and pod number was shown to be strongly related to grain yield (Devasirvatham et al. 2015). We observed this outcome where locations which experienced high likelihoods of frost were overall low yielding. Therefore, we demonstrated the importance of sowing date, and moisture availability in the form of PAWC and growing season rainfall on grain yield. The simulated yield values were similar to average yields reported by Dreccer et al. (2018) and in the National Variety Trials (https://www.nvtonline.com.au/). Overall, the influence of PAWC was fairly small (2.80 kg/ha, for each 1 mm increase in PAWC) and we conclude that growing season rainfall still has the strongest water influence on grain yield because it accounted for 5.79 kg/ha for each 1 mm increase.

In this study, a clear relationship between the cold temperature threshold (*T*_*C*_) and yield loss were quantified (Fig. 6), where the effect of *T*_*C*_, on yield loss ranged from − 12.3 to − 492 kg/ha. The differences in *T*_*C*_ detection are a summation of the diverse climatic and environmental conditions at the 95 locations. Therefore, we predict a trend appears where cold temperature thresholds are higher in northern and eastern areas and in inland areas compared to coastal areas (Fig. S4). The *T*_*C*_ seemed to be more related to latitude, longitude, temperature, and day length, but also to some extent to soil type, rainfall, and minimum temperature. We conclude that these factors can regulate and/or provide relief and minimise cold stress and frost-related yield losses. For all the three cultivars, the yield losses due to *T*_*C*_ detection decreased with delayed sowing. At some locations, presumably having high frost risk, such as Cunderdin (Western Australia), Roseworthy (South Australia), Horsham (Victoria), Walgett (New South Wales), and Dalby (Queensland); the *T*_*C*_ was detected and caused varying yield losses, while in other locations it was not detected. At Billa Billa in Queensland, early sown PBA HatTrick had the extreme *T*_*C*_ induced yield loss (Fig. 6). Thus, we recommended that growers at the diverse locations used in this study, understand the potential optimum sowing time for the applicable cultivars. Breeding for reproductive cold tolerance should remain a priority for the chickpea breeding programs.

## Conclusions

Our current study is consistent with the earlier finding of Chauhan et al. (2019) that soil moisture is a key driver of both flowering time and grain yield in chickpea. We demonstrated the importance of sowing date, moisture availability (PAWC), and growing season rainfall on chickpea grain yield. Early and late sowings were shown to result in yield losses, with intermediate sowing giving a yield advantage. Cold temperature threshold (*T*_*C*_) and its relationship with grain yield loss were quantified using data generated by the APSIM-chickpea model. Regions likely to experience high frost incidence were generally lower yielding. However, regions with relatively high *T*_*C*_ values may be at more risk of chilling-induced yield loss than might be suggested by the other climatic and soil data. This warrants further investigation. Our updated model is an improvement from the previous APSIM-chickpea version as it incorporates soil moisture, in addition to temperature and day length, and has been adequately parameterised for the cultivar PBA HatTrick. The ability of our model to simulate biomass accumulation, flowering time, and grain yield over a wide range of environmental conditions provides confidence that it can be applied across Australia to identify potential new growing areas.

## Supplementary Information

Below is the link to the electronic supplementary material.

Supplementary file1 (DOCX 2723 kb)
