# Supplementary material for: Modelling the effects of cold temperature during the reproductive stage on the yield of chickpea (Cicer arietinum L.)
Source: Int J Biometeorol. 2021 Oct 5;66(1):111–25. doi: 10.1007/s00484-021-02197-8 (PMC8727402; doi:10.1007/s00484-021-02197-8)
Supplement: Supplementary file 1 — Supplementary file1 (DOCX 2723 kb) [file 484_2021_2197_MOESM1_ESM.docx]

**Supplementary material**

**Appendix A. Supplementary data**

**Modelling the effects of cold temperature during the reproductive stage on the yield of chickpea**

Muhuddin Rajin Anwar, David J. Luckett, Yashvir S. Chauhan, H. L. Ip, Lancelot Maphosa, Marja Simpson, Annie Warren, Rosy Raman, Mark F. Richards, Georgina Pengilley, Kristy Hobson, Neroli Graham

**Supplemental Tables**

| **Table S1** Average minimum and maximum temperature (T, °C) for the period from the start of flowering to the start of podding in cultivar PBA HatTrick in field experiments where temperature was measured at canopy height. VIC= Victoria, NSW= New South Wales, QLD=Queensland | | | | |
| --- | --- | --- | --- | --- |
| **Location, State** | **Latitude/Longitude** | **Sowing date** | **Minimum T** | **Maximum T** |
| Horsham, VIC | -36.6697; 142.1731 | 16 May 2019 | 5.9 | 20.5 |
| Wagga Wagga, NSW | -35.0517; 147.3493 | 8 May 2018  28 May 20118 | 1.5  4.1 | 22.6  24.1 |
| Leeton, NSW | -34.5908; 146.3679 | 9 May 2019 | 3.2 | 21.0 |
| Tamworth, NSW | -31.0867; 150.8467 | 7 May 2018  12 Jun. 2018 | 4.8  6.3 | 20.5  24.8 |
| Breeza, NSW | -31.1781; 150.4240 | 15 May 2019 | 1.1 | 22.0 |
| Narrabri, NSW | -30.1946; 149.6089 | 13 May 2019 | 2.2 | 20.8 |
| Kingaroy, QLD | -26.5544; 151.8456 | 18 May 2019 | 3.5 | 20.9 |

| **Table S2** The APSIM-chickpea modified parameters of desi chickpea cultivar “PBA HatTrick”. TT = thermal time, HI = harvest index | | | | |
| --- | --- | --- | --- | --- |
| **Parameter** | **Units** | **Description** | **Range** | |
| x_pp_hi_incr | h | Photoperiod | 1 | 24 |
| y_hi_incr | 1/d | Rate of HI increase | 0.01 | 0.01 |
| x_hi_max_pot_stress |  | Average stress at flowering | 0 | 1 |
| y_hi_max_pot |  | Maximum harvest index potential | 0.4 | 0.4 |
| cum_vernal_days | d |  | 0 | 100 |
| tt_emerg_to_endjuv | °Cd | TT from emergence to end of juvenile phase | 690 | 690 |
| est_days_emerg_to_init |  | Estimated days from emergence to floral initiation | 83 | d |
| x_pp_endjuv_to_init | h | Photoperiod | 10.7 | 12 |
| y_tt_endjuv_to_init | °Cd | TT from end juvenile to floral initiation | 446 | 0 |
| x_pp_init_to_flower | h | Photoperiod | 1 | 24 |
| Y_tt_init_to_flower | °Cd | TT from initiation to flowering | 33 | 33 |
| x_pp_flower_to_start_grain | h | Photoperiod | 1 | 24 |
| y_tt_flower_to_start_grain | °Cd | TT from flowering to start grain fill | 450 | 450 |
| x_pp_start_to_end_grain | h | Photoperiod | 1 | 24 |
| y_tt_start_to_end_grain | °Cd | TT from start grain fill to end grain fill | 690 | 690 |
| tt_end_grain_to_maturity | °Cd | TT from end grain fill to maturity | 60 | 60 |
| tt_maturity_to_ripe | °Cd | TT from maturity to harvest ripe | 1 | 1 |
| x_stem_wt | g/plant | Stem weight | 0 | 10 |
| y_height | mm | Plant height | 0 | 800 |

| **Table S3** Site descriptions including soil types (Isbell 2016), plant available water capacity (PAWC), long-term average (1950-2019) rainfall, temperature, and frost incidence. A_O = growing season (April to October), MJJA = May to August, avT = mean temperature, maxT = maximum temperature, and minT = minimum temperature with coefficient of variation in parentheses (%). WA = Western Australia, SA = South Australia, QLD = Queensland, NSW = New South Wales, and VIC = Victoria. | | | | | | | | | | | | | |
| --- | --- | --- | --- | --- | --- | --- | --- | --- | --- | --- | --- | --- | --- |
|  |  |  |  |  |  |  |  | Annual average | | | | | |
|  |  |  |  |  | Sowing date | | | Rainfall | | Temperature (A_O) | | | Frost during MJJA  (day) |
| Sl. No. | Location  (State) | Latitude  Longitude | Soil type | PAWC  (mm) | Early | Mid | Late | Annual  (mm) | A_O  (mm) | avT  (°C) | MaxT  (°C) | MinT  (°C) |  |
| 1 | Dalwallinu  (WA) | -30.2772  116.6619 | Loamy duplex | 164 | 10-April to 30-April | 1-May to 21-May | 22-May to 11-June | 353  (25.6%) | 270  (26.9%) | 15.0  (4.11%) | 21.2  (3.93%) | 8.9  (6.44%) | 0.5  (188.82%) |
| 2 | Mingenew  (WA) | -29.1906  115.4414 | Kandosol | 245 | 10-April to 30-April | 1-May to 21-May | 22-May to 11-June | 378  (27.0%) | 321  (28.7%) | 16.4  (4.16%) | 22.9  (3.74%) | 10.0  (6.75%) | 0.2  (353.33%) |
| 3 | Pindar  (WA) | -28.4767  115.7897 | Sand | 195 | 10-April to 30-April | 1-May to 21-May | 22-May to 11-June | 295  (30.6%) | 222  (31.5%) | 16.2  (4.54%) | 22.9  (4.29%) | 9.6  (6.74%) | 0.5  (213.88%) |
| 4 | Three Springs  (WA) | -29.5339  115.7628 | Sandy clay | 268 | 10-April to 30-April | 1-May to 21-May | 22-May to 11-June | 367  (27.5% | 297  (27.6%) | 15.9  (4.24%) | 22.2  (3.97%) | 9.6  (6.35%) | 0.2  (353.33%) |
| 5 | Wongan Hills  (WA) | -30.8917  116.7186 | Loamy duplex | 140 | 10-April to 30-April | 1-May to 21-May | 22-May to 11-June | 387  (21.4%) | 309  (22.7%) | 14.7  (4.60%) | 20.8  (4.32%) | 8.7  (7.22%) | 1.1  (149.35%) |
| 6 | Morawa  (WA) | -29.1911  115.7867 | Kandosol | 254 | 10-April to 30-April | 1-May to 21-May | 22-May to 11-June | 370  (26.2%) | 305  (29.0%) | 15.5  (4.48%) | 21.9  (4.27%) | 9.1  (6.52%) | 0.6  (178.11%) |
| 7 | Yarragadee  (WA) | -29.0767  115.4092 | Chromosol | 199 | 10-April to 30-April | 1-May to 21-May | 22-May to 11-June | 338  (28.1%) | 288  (29.9%) | 16.6  (4.13%) | 23.0  (3.77%) | 10.2  (6.39%) | 0.1  (432.57%) |
| 8 | Eradu  (WA) | -28.6936  115.0408 | Loamy duplex | 96 | 10-April to 30-April | 1-May to 21-May | 22-May to 11-June | 357  (28.8%) | 308  (29.2%) | 16.5  (4.17%) | 22.7  (3.82%) | 10.3  (6.18%) | 0.1  (363.16%) |
| 9 | Badgingarra  (WA) | -30.3903  115.5039 | Sand duplex | 113 | 10-April to 30-April | 1-May to 21-May | 22-May to 11-June | 548  (22.8%) | 482  (22.4%) | 15.3  (3.91%) | 21.1  (3.46%) | 9.5  (6.38%) | 0.1  (587.31%) |
| 10 | Cunderdin  (WA) | -31.6494  117.2331 | Sandy duplex | 112 | 10-April to 30-April | 1-May to 21-May | 22-May to 11-June | 357  (24.3%) | 281  (26.5%) | 14.3  (4.03%) | 20.6  (4.11%) | 8.1  (10.12%) | 4.0  (146.21%) |
| 11 | Dowerin  (WA) | -31.1936  117.0311 | Loamy duplex | 119 | 10-April to 30-April | 1-May to 21-May | 22-May to 11-June | 351  (23.4%) | 270  (24.2%) | 14.3  (4.19%) | 20.4  (4.29%) | 8.1  (7.28%) | 2.5  (121.75%) |
| 12 | Kellerberrin  (WA) | -31.6183  117.7217 | Dermosol | 237 | 10-April to 30-April | 1-May to 21-May | 22-May to 11-June | 321  27.2%) | 240  (28.3%) | 14.1  (4.16%) | 20.5  (4.33%) | 7.6  (10.23%) | 7.9  (87.01%) |
| 13 | Merredin  (WA) | -31.4756  118.2789 | Dermosol | 169 | 10-April to 30-April | 1-May to 21-May | 22-May to 11-June | 324  (23.9%) | 237  (23.7%) | 14.0  (5.43%) | 20.2  (4.77%) | 7.8  (9.03%) | 8.2  (53.85%) |
| 14 | Williams  (WA) | -33.0269  116.8792 | Sandy duplex | 131 | 10-April to 30-April | 1-May to 21-May | 22-May to 11-June | 513  (22.5%) | 421  (22.9%) | 12.5  (4.78%) | 18.4  (4.11%) | 6.6  (10.50%) | 8.7  (66.40%) |
| 15 | Bodallin  (WA) | -31.33  118.9672 | Calcarosol | 101 | 10-April to 30-April | 1-May to 21-May | 22-May to 11-June | 328  (26.6%) | 233  (28.7%) | 13.7  (4.71%) | 20.2  (4.36%) | 7.2  (8.58%) | 10.5  (49.34%) |
| 16 | Cummins  (SA) | -34.2644  135.7266 | Clay loam | 153 | 1-May to 21-May | 22-May to 11-June | 12-June to 2-July | 410  24.2%) | 331  (25.5%) | 13.7  (3.25%) | 18.7  (3.41%) | 8.7  (6.53%) | 0.4  (242.03%) |
| 17 | Rudall  (SA) | -33.5883  136.2233 | Loamy duplex | 170 | 1-May to 21-May | 22-May to 11-June | 12-June to 2-July | 354  (27.8%) | 263  (30.4%) | 14.0  (3.76%) | 19.5  (3.87%) | 8.4  (5.48%) | 0.2  (270.32%) |
| 18 | Lock  (SA) | -33.5676  135.7561 | Sandy clay | 104 | 1-May to 21-May | 22-May to 11-June | 12-June to 2-July | 376  (24.8%) | 289  (27.6%) | 13.7  (3.85%) | 19.6  (3.83%) | 7.8  (6.87%) | 2.2  (132.80%) |
| 19 | Kielpa  (SA) | -33.69  136.2683 | Sandy duplex | 164 | 1-May to 21-May | 22-May to 11-June | 12-June to 2-July | 336  (27.4%) | 248  (29.4%) | 14.2  (3.65) | 19.5  (3.79%) | 8.8  (5.13% | 0.1  (346.83%) |
| 20 | Yeelanna  (SA) | -34.1317  135.7301 | Loamy duplex | 78 | 1-May to 21-May | 22-May to 11-June | 12-June to 2-July | 415  (27.5%) | 331  (27.7%) | 13.6  (3.34%) | 18.7  (3.53%) | 8.5  (6.18%) | 0.5  (229.16%) |
| 21 | Cleve  (SA) | -33.7081  136.5026 | Sandy clay | 98 | 1-May to 21-May | 22-May to 11-June | 12-June to 2-July | 371  (26.5$) | 267  (29.5%) | 13.8  (4.20) | 18.8  (4.06%) | 8.8  (6.11%) | 0.1  (422.21%) |
| 22 | Brinkworth  (SA) | -33.6903  138.4041 | Calcarosol clay loam | 207 | 1-May to 21-May | 22-May to 11-June | 12-June to 2-July | 415  (25.3%) | 303  (28.3%) | 12.9  (3.58%) | 18.8  (3.95%) | 6.9  (7.43%) | 7.9  (74.89%) |
| 23 | Mintaro  (SA) | -33.9136  138.7195 | Vertosol black clay | 164 | 1-May to 21-May | 22-May to 11-June | 12-June to 2-July | 590  (24.4%) | 454  (28.4%) | 11.4  (4.23%) | 16.8  (4.38%) | 5.9  (8.03%) | 12.9  (45.30%) |
| 24 | Snowtown  (SA) | -33.7844  138.2133 | Calcarosol clay loam | 176 | 1-May to 21-May | 22-May to 11-June | 12-June to 2-July | 418  (27.5%) | 310  (28.7%) | 13.0  (4.55%) | 19.1  (4.38%) | 7.0  (9.52%) | 9.1  (67.20%) |
| 25 | Hilltown  (SA) | -33.6867  138.6378 | Chromosol red | 123 | 1-May to 21-May | 22-May to 11-June | 12-June to 2-July | 465  (24.8%) | 357  (28.9%) | 11.7  (4.05%) | 17.5  (4.32%) | 6.0  (7.56%) | 14.2  (42.43%) |
| 26 | Paskeville  (SA) | -34.0402  137.9004 | Dermosol Red | 127 | 1-May to 21-May | 22-May to 11-June | 12-June to 2-July | 384  (27.0%) | 293  (28.6%) | 13.2  (3.44%) | 18.6  (3.66%) | 7.8  (6.14%) | 2.0  (135.58%) |
| 27 | Sandilands  (SA) | -34.5201  137.7733 | Sandy clay | 181 | 1-May to 21-May | 22-May to 11-June | 12-June to 2-July | 409  (22.4%) | 317  (23.4%) | 13.3  (3.76%) | 17.9  (3.75%) | 8.7  (5.53%) | 0.2  (370.04%) |
| 28 | Mallala  (SA) | -34.4382  138.5115 | Calcarosol | 126 | 1-May to 21-May | 22-May to 11-June | 12-June to 2-July | 393  (24.7%) | 289  (25.9%) | 13.6  (3.36%) | 19.0  (3.83%) | 8.2  (5.56%) | 1.4  (141.85%) |
| 29 | Roseworthy  (SA) | -34.5106  138.6763 | Sodosol Sandy clay | 126 | 1-May to 21-May | 22-May to 11-June | 12-June to 2-July | 422  (24.2%) | 313  (26.4%) | 13.3  (3.80%) | 18.8  (4.48%) | 7.9  (8.22%) | 3.1  (124.48%) |
| 30 | Riverton  (SA) | -34.1579  138.7452 | Sandy clay | 273 | 1-May to 21-May | 22-May to 11-June | 12-June to 2-July | 518  (24.8%) | 390  (26.9%) | 13.2  (3.56) | 19.0  (3.89%) | 7.4  (6.61%) | 4.0  (91.54) |
| 31 | Capella  (QLD) | -23.0856  148.0236 | Vertosol | 146 | 15-April to 5-May | 6-May to 26-May | 27-May to 16-June | 604  (34.5%) | 196  (58.1%) | 19.2  (3.87%) | 26.6  (3.16%) | 11.8  (8.02%) | 1.5  (150.39%) |
| 32 | Clermont  (QLD) | -22.825  147.6408 | Vertosol | 261 | 15-April to 5-May | 6-May to 26-May | 27-May to 16-June | 643  (36.8%) | 193  (58.3%) | 19.2  (3.77%) | 26.8  (3.27%) | 11.5  (8.25%) | 4.1  (99.56%) |
| 33 | Duaringa  (QLD) | -23.7139  149.6725 | Vertosol | 287 | 15-April to 5-May | 6-May to 26-May | 27-May to 16-June | 717  (34.8%) | 235  (51.4%) | 19.4  (3.39%) | 26.6  (2.95%) | 12.1  (6.78%) | 0.9  (169.38%) |
| 34 | Gindie  (QLD) | -23.7  148.1 | Vertosol | 140 | 15-April to 5-May | 6-May to 26-May | 27-May to 16-June | 592  (39.8%) | 194  (61.4%) | 19.1  (4.01) | 26.6  (3.13%) | 11.7  (8.47%) | 1.7  (161.43%) |
| 35 | Orion  (QLD) | -24.2644  148.3825 | Vertosol | 137 | 15-April to 5-May | 6-May to 26-May | 27-May to 16-June | 670  (42.9%) | 222  (61.5%) | 18.5  (3.78%) | 26.3  (3.15%) | 10.7  (8.33%) | 4.0  (112.44%) |
| 36 | Rolleston  (QLD) | -24.4619  148.6261 | Vertosol | 137 | 15-April to 5-May | 6-May to 26-May | 27-May to 16-June | 628  (37.6%) | 226  (58.7%) | 18.1  (3.60%) | 26.0  (3.34%) | 10.1  (8.33%) | 6.6  (84.00%) |
| 37 | Springsure  (QLD) | -24.123  148.0856 | Vertosol | 159 | 15-April to 5-May | 6-May to 26-May | 27-May to 16-June | 715  (38.8%) | 241  (54.4%) | 17.9  (5.30%) | 25.4  (3.82%) | 10.4  (11.54%) | 6.7  (83.85%) |
| 38 | Emerald  (QLD) | -23.5  148.15 | Vertosol | 287 | 15-April to 5-May | 6-May to 26-May | 27-May to 16-June | 618  (35.8%) | 202  (59.3%) | 19.4  (3.99%) | 26.7  (3.14%) | 12.1  (8.20%) | 1.2  (174.59%) |
| 39 | Banana  (QLD) | -24.4719  150.1292 | Vertosol | 189 | 15-April to 5-May | 6-May to 26-May | 27-May to 16-June | 629  (28.7%) | 222  (48.5%) | 18.2  (3.68%) | 26.0  (3.43%) | 10.4  (8.20%) | 4.8  (92.69%) |
| 40 | Baralaba  (QLD) | -24.1819  149.8117 | Vertosol | 156 | 15-April to 5-May | 6-May to 26-May | 27-May to 16-June | 706  (32.1%) | 244  (46.6%) | 19.0  (3.58%) | 26.7  (3.20%) | 11.4  (7.23%) | 1.9  (125.74%) |
| 41 | Biloela  (QLD) | -24.3789  150.5164 | Sodosol | 185 | 15-April to 5-May | 6-May to 26-May | 27-May to 16-June | 646  (28.6%) | 227  (41.4%) | 18.0  (3.98%) | 25.9  (3.30%) | 10.0  (10.58%) | 8.1  (84.26%) |
| 42 | Gayndah  (QLD) | -25.6258  151.6094 | Vertosol | 213 | 30-April to 20-May | 21-May to 10-June | 11-June to 1-July | 742  (27.6%) | 277  (40.7%) | 18.1  (3.77%) | 25.6  (3.14%) | 10.6  (8.90%) | 4.3  (99.17%) |
| 43 | Moura  (QLD) | -24.5722  149.9694 | Vertosol | 137 | 15-April to 5-May | 6-May to 26-May | 27-May to 16-June | 663  (31.0%) | 239  (46.4%) | 18.5  (3.58%) | 26.4  (3.41%) | 10.7  (7.84%) | 3.7  (100.86%) |
| 44 | Theodore  (QLD) | -24.9503  150.0725 | Vertosol | 129 | 15-April to 5-May | 6-May to 26-May | 27-May to 16-June | 680  (31.8%) | 255  (49.0%) | 18.1  (3.65)% | 25.9  (3.73%) | 10.2  (8.24%) | 6.2  (78.51%) |
| 45 | Kingaroy  (QLD) | -26.5544  151.8456 | Ferrosol | 100 | 30-April to 20-May | 21-May to 10-June | 11-June to 1-July | 771  (25.9%) | 305  (37.7%) | 15.1  (4.38%) | 22.3  (3.79%) | 7.9  (12.52%) | 20.5  (48.56%) |
| 46 | Kumbia  (QLD) | -26.6894  151.655 | Ferrosol | 100 | 30-April to 20-May | 21-May to 10-June | 11-June to 1-July | 782  (25.3%) | 318  (35.8%) | 14.8  (4.04%) | 21.9  (3.83%) | 7.7  (11.16%) | 20.8  (43.08%) |
| 47 | Cecilplains  (QLD) | -27.5331  151.2025 | Vertosol | 168 | 30-April to 20-May | 21-May to 10-June | 11-June to 1-July | 640  (26.1%) | 268  (44.6%) | 15.3  (4.02%) | 22.7  (4.03%) | 7.9  (9.79%) | 19.8  (42.79%) |
| 48 | Condamine Plains (QLD) | -27.7233  151.2869 | Vertosol | 234 | 30-April to 20-May | 21-May to 10-June | 11-June to 1-July | 645  (27.8%) | 266  (44.9%) | 15.1  (4.11%) | 22.6  (3.99%) | 7.7  (9.95%) | 20.6  (40.79%) |
| 49 | Goondiwindi  (QLD) | -28.5481  150.3075 | Vertosol | 174 | 30-April to 20-May | 21-May to 10-June | 11-June to 1-July | 616  (26.5%) | 269  (45.8%) | 16.1  (3.77%) | 23.1  (4.42%) | 9.0  (9.09%) | 9.0  (72.77%) |
| 50 | Hermitage  (QLD) | -28.2061  152.1003 | Vertosol | 216 | 30-April to 20-May | 21-May to 10-June | 11-June to 1-July | 702  (24.0%) | 292  (39.0%) | 13.6  (4.75%) | 20.5  (4.61%) | 6.6  (14.12%) | 30.1  (40.77%) |
| 51 | Jimbour  (QLD) | -26.9636  151.2158 | Vertosol | 254 | 30-April to 20-May | 21-May to 10-June | 11-June to 1-July | 632  (27.0%) | 258  (40.6%) | 15.6  (3.87%) | 23.1  (4.00%) | 8.1  (10.24%) | 19.1  (47.85%) |
| 52 | Warwick  (QLD) | -28.2167  152.0333 | Vertosol | 245 | 30-April to 20-May | 21-May to 10-June | 11-June to 1-July | 675  (26.0%) | 284  (42.8%) | 13.8  (4.31%) | 20.8  (4.25%) | 6.8  (11.56%) | 27.4  (35.88%) |
| 53 | Warra  (QLD) | -26.9308  150.925 | Vertosol | 207 | 30-April to 20-May | 21-May to 10-June | 11-June to 1-July | 653  (30.0%) | 269  (44.5%) | 15.9  (3.91%) | 23.4  (4.01%) | 8.3  (10.03%) | 18.3  (48.95%) |
| 54 | Brookstead  (QLD) | -27.7578  151.4483 | Vertosol | 287 | 30-April to 20-May | 21-May to 10-June | 11-June to 1-July | 641  (25.9%) | 263  (42.1%) | 15.0  (4.11%) | 22.4  (3.93%) | 7.6  (10.08%) | 21.2  (39.98%) |
| 55 | Billa Billa  (QLD) | -28.17  150.4503 | Vertosol | 183 | 30-April to 20-May | 21-May to 10-June | 11-June to 1-July | 550  (28.5%) | 238  (43.8%) | 15.8  (4.29%) | 23.2  (4.27%) | 8.5  (9.29%) | 13.5  (56.42%) |
| 56 | Dalby  (QLD) | -27.1605  151.2634 | Vertosol | 200 | 30-April to 20-May | 21-May to 10-June | 11-June to 1-July | 626  (25.1%) | 257  (41.7%) | 15.6  (3.83%) | 23.1  (4.03%) | 8.2  (10.12%) | 18.9  (50.57%) |
| 57 | Meandarra  (QLD) | -27.3236  149.8828 | Vertosol | 186 | 30-April to 20-May | 21-May to 10-June | 11-June to 1-July | 592  (30.2%) | 255  (44.5%) | 16.1  (4.02%) | 23.6  (3.98%) | 8.5  (9.64%) | 16.9  (48.16%) |
| 58 | Miles  (QLD) | -26.6581  150.1844 | Vertosol | 285 | 30-April to 20-May | 21-May to 10-June | 11-June to 1-July | 643  (28.6%) | 260  (48.4%) | 16.0  (4.55%) | 23.8  (4.23%) | 8.3  (11.15%) | 21.4  (45.05%) |
| 59 | Thallon  (QLD) | -28.6342  148.8681 | Vertosol | 186 | 30-April to 20-May | 21-May to 10-June | 11-June to 1-July | 525  (32.2%) | 226  (45.8%) | 16.3  (3.89%) | 23.6  (4.15%) | 9.0  (8.34%) | 8.0  (67.30%) |
| 60 | Bungunya  (QLD) | -28.4278  149.6533 | Sodosol | 156 | 30-April to 20-May | 21-May to 10-June | 11-June to 1-July | 538  (27.7%) | 232  (46.3%) | 16.2  (3.78%) | 23.5  (4.15%) | 9.0  (8.24%) | 9.2  (64.51%) |
| 61 | Condamine  (QLD) | -26.9269  150.1419 | Vertosol | 285 | 30-April to 20-May | 21-May to 10-June | 11-June to 1-July | 611  (29.4$) | 256  (48.5%) | 16.1  (4.19%) | 23.8  (4.06%) | 8.4  (10.17%) | 18.6  (47.60%) |
| 62 | Roma  (QLD) | -26.5719  148.7897 | Vertosol | 119 | 30-April to 20-May | 21-May to 10-June | 11-June to 1-July | 604  (31.9%) | 247  (46.3%) | 16.4  (3.88%) | 24.3  (3.63%) | 8.6  (11.65%) | 20.4  (53.75%) |
| 63 | St. George  (QLD) | -28.0361  148.5814 | Vertosol | 191 | 30-April to 20-May | 21-May to 10-June | 11-June to 1-July | 527  (32.7%) | 227  (46.3%) | 16.7  (3.52%) | 23.8  (3.58%) | 9.6  (8.75%) | 6.9  (89.65%) |
| 64 | Condobolin  (NSW) | -33.0664  147.2283 | Sodosol | 143 | 10-May to 30-May | 31-May to 21-June | 22-June to 11-July | 456  (34.5%) | 245  (41.8%) | 12.9  (4.38%) | 19.6  (4.45%) | 6.2  (11.54%) | 26.8  (43.47%) |
| 65 | Trangie  (NSW) | -31.9861  147.9489 | Sandy clay loam | 141 | 10-May to 30-May | 31-May to 21-June | 22-June to 11-July | 509  (38.2%) | 264  (45.6%) | 13.6  (4.93%) | 20.2  (4.99%) | 6.9  (9.99%) | 19.1  (41.18%) |
| 66 | Coonamble  (NSW) | -31.000  148.4333 | Sandy clay | 181 | 30-April to 20-May | 21-May to 10-June | 11-June to 1-July | 523  (34.6%) | 254  (47.2%) | 14.5  (3.68%) | 21.6  (3.95%) | 7.3  (9.16%) | 15.3  (46.87%) |
| 67 | Mungindi  (NSW) | -28.9786  148.9899 | Vertosol | 206 | 30-April to 20-May | 21-May to 10-June | 11-June to 1-July | 521  (31.6%) | 225  (45.7%) | 16.2  (4.40%) | 23.7  (4.58%) | 8.7  (9.06%) | 9.9  (62.37%) |
| 68 | Walgett  (NSW) | -30.0236  148.1218 | Vertosol | 191 | 10-May to 30-May | 31-May to 21-June | 22-June to 11-July | 481  (36.0%) | 229  (47.6%) | 15.6  (3.36%) | 23.0  (3.98%) | 8.2  (9.91%) | 13.0  (62.81) |
| 69 | Bellata  (NSW) | -29.9182  149.7919 | Vertosol | 198 | 10-May to 30-May | 31-May to 21-June | 22-June to 11-July | 634  (27.0%) | 284  (39.3%) | 14.9  (4.17%) | 22.3  (4.08%) | 7.5  (10.32%) | 17.7  (46.21%) |
| 70 | Weewaa  (NSW) | -30.2083  149.5967 | Vertosol | 194 | 10-May to 30-May | 31-May to 21-June | 22-June to 11-July | 601  (29.2%) | 278  (42.6%) | 15.0  (4.11%) | 22.5  (3.98%) | 7.6  (10.15%) | 15.9  (50.55%) |
| 71 | Narrabri  (NSW) | -30.2552  149.6789 | Clay Loam | 227 | 10-May to 30-May | 31-May to 21-June | 22-June to 11-July | 610  (29.7%) | 281  (41.1%) | 15.0  (4.15%) | 22.4  (3.98%) | 7.5  (10.31%) | 16.6  (49.93%) |
| 72 | Moree  (NSW) | -29.500  149.900 | Vertosol | 210 | 10-May to 30-May | 31-May to 21-June | 22-June to 11-July | 593  (28.2%) | 259  (42.6%) | 15.3  (4.32%) | 22.4  (4.48%) | 8.1  (10.24%) | 16.1  (47.43%) |
| 73 | Tulloona  (NSW) | -28.8695  150.0894 | Vertosol | 203 | 30-April to 20-May | 21-May to 10-June | 11-June to 1-July | 597  (27.8%) | 257  (44.5%) | 15.7  (3.98%) | 22.9  (4.27%) | 8.4  (8.79%) | 12.1  (59.11%) |
| 74 | Breeza  (NSW) | -31.2475  150.4632 | Vertosol | 204 | 30-April to 20-May | 21-May to 10-June | 11-June to 1-July | 648  (26.7%) | 301  (41.2%) | 13.9  (3.45%) | 20.9  (4.26%) | 6.9  (10.05%) | 19.2  (45.33%) |
| 75 | Gunnedah  (NSW) | -30.9841  150.254 | Vertosol | 204 | 10-May to 30-May | 31-May to 21-June | 22-June to 11-July | 648  (26.5%) | 305  (37.7%) | 14.4  (4.02%) | 21.2  (4.34%) | 7.5  (10.80%) | 16.8  (46.22%) |
| 76 | Quirindi  (NSW) | -31.5086  150.6793 | Vertosol | 245 | 10-May to 30-May | 31-May to 21-June | 22-June to 11-July | 683  (24.8%) | 329  (37.5%) | 12.9  (4.28%) | 20.3  (4.71%) | 5.5  (14.40%) | 36.2  (34.01%) |
| 77 | Tamworth  (NSW) | -31.0867  150.8467 | Vertosol | 204 | 10-May to 30-May | 31-May to 21-June | 22-June to 11-July | 670  (26.0%) | 318  (35.0%) | 13.2  (3.95%) | 20.1  (5.13%) | 6.3  (13.50%) | 25.0  (46.64%) |
| 78 | Wellington  (NSW) | -32.5059  148.9708 | Sandy clay loam | 101 | 30-April to 20-May | 21-May to 10-June | 11-June to 1-July | 615  (31.3%) | 327  (39.3%) | 12.7  (4.21%) | 19.0  (5.89%) | 6.4  (12.94%) | 22.4  (45.04%) |
| 79 | Wagga Wagga  (NSW) | -35.0517  147.3493 | Kandosol | 110 | 10-May to 30-May | 31-May to 21-June | 22-June to 11-July | 549  (29.3%) | 334  (34.9%) | 11.6  (4.12%) | 17.4  (5.30%) | 5.8  (11.76%) | 22.6  (43.57%) |
| 80 | Yenda  (NSW) | -34.2502  146.1897 | Sandy Loam | 165 | 10-May to 30-May | 31-May to 21-June | 22-June to 11-July | 438  (32.4%) | 258  (36.6%) | 12.8  (3.98%) | 19.2  (4.35%) | 6.5  (9.20%) | 17.9  (48.22%) |
| 81 | Nyah West  (VIC) | -35.1833  143.3333 | Sandy duplex | 142 | 1-May to 21-May | 22-May to 11-June | 12-June to 2-July | 353  (32.9%) | 222  (37.2%) | 12.6  (3.48%) | 18.7  (4.20%) | 6.5  (8.49%) | 9.4  (70.35%) |
| 82 | Ouyen  (VIC) | -35.0682  142.3125 | Loamy duplex | 214 | 1-May to 21-May | 22-May to 11-June | 12-June to 2-July | 337  (32.6%) | 213  (36.1%) | 13.0  (3.73%) | 19.3  (4.07%) | 6.8  (6.66%) | 7.5  (61.16%) |
| 83 | Birchip  (VIC) | -35.9244  142.851 | Clay loam | 195 | 1-May to 21-May | 22-May to 11-June | 12-June to 2-July | 364  (28.9%) | 239  (34.9%) | 12.1  (3.54%) | 18.1  (4.44%) | 6.2  (8.53%) | 9.7  (68.42%) |
| 84 | Labert  (VIC) | -35.5405  143.5286 | Sandy clay | 226 | 1-May to 21-May | 22-May to 11-June | 12-June to 2-July | 338  (30.8%) | 217  (35.3%) | 12.4  (3.40%) | 18.4  (4.29%) | 6.5  (8.90%) | 8.9  (78.37%) |
| 85 | Sea Lake  (VIC) | -35.5046  142.8493 | Loamy duplex | 78 | 1-May to 21-May | 22-May to 11-June | 12-June to 2-July | 340  (33.0%) | 215  (39.4%) | 12.6  (3.43%) | 18.8  (4.23%) | 6.5  (8.08%) | 8.3  (72.17%) |
| 86 | Jil Jil  (VIC) | -35.8167  143.0333 | Sandy clay | 255 | 1-May to 21-May | 22-May to 11-June | 12-June to 2-July | 350  (29.7%) | 232  (36.3%) | 12.3  (3.51%) | 18.2  (4.43%) | 6.3  (8.57%) | 9.3  (73.13%) |
| 87 | Brim  (VIC) | -36.0667  142.4167 | Clay loam | 183 | 1-May to 21-May | 22-May to 11-June | 12-June to 2-July | 358  (29.1%) | 242  (33.2%) | 12.1  (3.46%) | 18.1  (4.20%) | 6.0  (8.52%) | 10.2  (63.98%) |
| 88 | Warracknabeal  (VIC) | -36.2705  142.2165 | Clay loam | 216 | 1-May to 21-May | 22-May to 11-June | 12-June to 2-July | 391  (27.4%) | 267  (30.7%) | 11.8  (3.51%) | 17.7  (4.17%) | 5.8  (9.10%) | 11.3  (60.47%) |
| 89 | Kalkee  (VIC) | -36.5167  142.1667 | Black Vertosols | 179 | 1-May to 21-May | 22-May to 11-June | 12-June to 2-July | 411  (27.4%) | 286  (31.3%) | 11.4  (3.59%) | 17.3  (4.23%) | 5.5  (10.95%) | 13.6  (62.74%) |
| 90 | Gymbowen  (VIC) | -36.7181  141.4729 | Clay loam | 174 | 1-May to 21-May | 22-May to 11-June | 12-June to 2-July | 497  (24.1%) | 365  (28.3%) | 11.1  (4.27%) | 16.6  (4.20%) | 5.5  (10.07%) | 12.9  (53.02%) |
| 91 | St Arnaud  (VIC) | -36.6175  143.2644 | Clay | 202 | 1-May to 21-May | 22-May to 11-June | 12-June to 2-July | 516  (28.6%) | 358  (32.9%) | 10.8  (4.16%) | 16.2  (4.87%) | 5.5  (8.11%) | 14.9  (46.77%) |
| 92 | Goroke  (VIC) | -36.700  141.400 | Sodosols | 143 | 1-May to 21-May | 22-May to 11-June | 12-June to 2-July | 498  (22.8%) | 365  (27.1%) | 11.1  (4.27%) | 16.7  (4.19%) | 5.6  (10.00%) | 12.5  (53.64%) |
| 93 | Apsley  (VIC) | -36.9681  141.0834 | Sandy duplex | 158 | 1-May to 21-May | 22-May to 11-June | 12-June to 2-July | 567  (20.8%) | 424  (24.4%) | 11.3  (4.23%) | 16.6  (4.05%) | 6.0  (9.25%) | 10.3  (59.12$) |
| 94 | Rupanyup  (VIC) | -36.6287  142.6321 | Vertosols | 223 | 1-May to 21-May | 22-May to 11-June | 12-June to 2-July | 428  (26.2%) | 296  (30.3%) | 11.4  (3.71%) | 16.9  (4.52%) | 5.9  (8.89%) | 10.1  (63.95%) |
| 95 | Horsham  (VIC) | -36.6697  142.1731 | Vertosols | 249 | 1-May to 21-May | 22-May to 11-June | 12-June to 2-July | 418  (26.8%) | 291  (31.5%) | 11.3  (3.76%) | 17.1  (4.25%) | 5.4  (13.96%) | 15.3  (66.24%) |

| **Table S4** ANOVA table for linear model between flowering time and explanatory variables. These variables are cultivar, sowing time, State comprising 95 locations, plant available water holding capacity (PAWC in mm) and growing season rainfall (mm), with two-way interactions between cultivar, sowing time and State. F = F test statistics, p = level of significance, Effect = estimated coefficient, SS = sum of squares, df = degrees of freedom. QLD=Queensland, SA= South Australia, VIC= Victoria, WA=Western Australia | | | | |
| --- | --- | --- | --- | --- |
|  | F | p | Effect | p |
| Cultivar | 662.08 | < 001 |  |  |
| PBA HatTrick |  |  | 1.33 | < 001 |
| PBA Seamer |  |  | -1.39 | <.001 |
| Sowing date | 45455.48 | <.001 |  |  |
| Mid |  |  | 18.21 | <.001 |
| Late |  |  | 34.62 | <.001 |
| State | 25667.37 | <.001 |  |  |
| QLD |  |  | -15.52 | < 001 |
| SA |  |  | -5.32 | <.001 |
| VIC |  |  | 6.55 | <.001 |
| WA |  |  | -21.36 | <.001 |
| PAWC | 1367.60 | <.001 | -0.02 | <.001 |
| Growing season rainfall | 38261.53 | <.001 | 0.16 | <.001 |
| Cultivar x Sowing date | 1.83 | 0.117 |  |  |
| PBA HatTrick x Mid |  |  | 0.18 | 0.497 |
| PBA HatTrick x Late |  |  | 0.15 | 0.578 |
| PBA Seamer x Mid |  |  | -0.38 | 0.143 |
| PBA Seamer x Late |  |  | -0.14 | 0.595 |
| Cultivar x State | 3.57 | <.001 |  |  |
| PBA HatTrick x QLD |  |  | -0.28 | 0.391 |
| PBA HatTrick x SA |  |  | 0.13 | 0.754 |
| PBA HatTrick x VIC |  |  | 0.59 | 0.132 |
| PBA HatTrick x WA |  |  | 0.19 | 0.631 |
| PBA Seamer x QLD |  |  | 0.32 | 0.314 |
| PBA Seamer x SA |  |  | -0.33 | 0.418 |
| PBA Seamer x VIC |  |  | -0.41 | 0.287 |
| PBA Seamer x WA |  |  | 0.01 | 0.983 |
| Sowing date x State | 42.13 | <.001 |  |  |
| Mid x QLD |  |  | -1.29 | < 001 |
| Mid x SA |  |  | -0.43 | 0.286 |
| Mid x VIC |  |  | 0.08 | 0.842 |
| Mid x WA |  |  | 0.35 | 0.370 |
| Late x QLD |  |  | -1.06 | < 001 |
| Late x SA |  |  | 2.92 | < 001 |
| Late x VIC |  |  | 2.61 | < 001 |
| Late x WA |  |  | -1.82 | <.001 |
| Residuals SS = 12137948 on 78049 df. | |  |  |  |

| **Table S5** ANOVA table for linear model between chickpea yield and explanatory variables. These variables are cultivar, sowing time, State comprising 95 locations, plant available water holding capacity (PAWC in mm) and growing season rainfall (mm), with two-way interactions between cultivar, sowing time and State. F = F test statistics, p = level of significance, Effect = estimated coefficient, SS = sum of squares, df = degrees of freedom. QLD=Queensland, SA= South Australia, VIC= Victoria, WA=Western Australia | | | | |
| --- | --- | --- | --- | --- |
|  | F | p | Effect | p |
| Cultivar | 246.11 | <.001 |  |  |
| PBA HatTrick |  |  | -149.06 | <.001 |
| PBA Seamer |  |  | -136.92 | <.001 |
| Sowing date | 584.28 | <.001 |  |  |
| Mid |  |  | -101.50 | <.001 |
| Late |  |  | -211.09 | <.001 |
| State | 2982.77 | <.001 |  |  |
| QLD |  |  | -17.98 | 0.397 |
| SA |  |  | 430.24 | <.001 |
| VIC |  |  | 236.14 | <.001 |
| WA |  |  | 760.87 | <.001 |
| PAWC | 1431.42 | <.001 | 2.80 | <.001 |
| Growing season rainfall | 9987.06 | <.001 | 5.79 | <.001 |
| Cultivar x Sowing date | 23.75 | <.001 |  |  |
| PBA HatTrick x Mid |  |  | 84.86 | <.001 |
| PBA HatTrick x Late |  |  | 144.42 | <.001 |
| PBA Seamer x Mid |  |  | 98.26 | <.001 |
| PBA Seamer x Late |  |  | 175.21 | <.001 |
| Cultivar x State | 36.81 | <.001 |  |  |
| PBA HatTrick x QLD |  |  | 20.79 | 0.364 |
| PBA HatTrick x SA |  |  | 29.52 | 0.197 |
| PBA HatTrick x VIC |  |  | -181.48 | <.001 |
| PBA HatTrick x WA |  |  | -215.79 | <.001 |
| PBA Seamer x QLD |  |  | -180.78 | <.001 |
| PBA Seamer x SA |  |  | -208.69 | <.001 |
| PBA Seamer x VIC |  |  | -228.25 | <.001 |
| PBA Seamer x WA |  |  | -259.80 | <.001 |
| Sowing date x State | 18.35 | <.001 |  |  |
| Mid x QLD |  |  | 11.33 | 0.621 |
| Mid x SA |  |  | 4.10 | 0.880 |
| Mid x VIC |  |  | 54.22 | 0.046 |
| Mid x WA |  |  | -172.10 | <.001 |
| Late x QLD |  |  | 10.60 | 0.643 |
| Late x SA |  |  | 38.23 | 0.160 |
| Late x VIC |  |  | 85.04 | 0.002 |
| Late x WA |  |  | -207.56 | <.001 |
| Residuals SS = 36906000000 on 59819 df. | |  |  |  |

| **Table S6** Geospatial criteria that were used in multi-criteria analysis (Chen et al. 2010) for delineating chickpea land suitability across the cropping regions of Australia. To determine a weighting of the criteria the analytical hierarchy process was applied (Saaty 1980). Suitability classes (very suitable, suitable, moderate suitable, low suitable, and unsuitable) are based on FAO descriptions (<http://www.fao.org/3/x5310e/x5310e00.htm>). The climate data used for the analysis of the climatic factors, was accessed through the NCI (2020) at a spatial resolution of 0.01° (approx. 1 km). Data for pH was derived from the Soil and Landscape Grid of Australia (<https://www.clw.csiro.au/aclep/soilandlandscapegrid/>) and the data for slope was generated from the Digital Elevation Model developed by Geoscience Australia (<https://www.ga.gov.au/scientific-topics/national-location-information/digital-elevation-data>). | | | | | | |
| --- | --- | --- | --- | --- | --- | --- |
|  |  | **Desi chickpea suitability categories** | | | | |
| **Geospatial data input** | **Weighting**  **(total 100%)** | **5**  **very suitable** | **4**  **suitable** | **3**  **moderate suitable** | **2**  **low suitable** | **1**  **unsuitable** |
| **Climate factors** | **0.91** |  |  |  |  |  |
| Mean annual rainfall  (mm) | 0.02 | 650-900 | 450-650 | 350-450 | 300-350 | <300  >900 |
| Growing season rainfall  (April−October) (mm) | 0.25 | 350–450 | 350-300 | 250-300 | 250-200  450-550 | <200  >550 |
| Mean temperature (T)  (April−October) (°C) | 0.03 | 17-25 | 15-17 | 12-15 | 12-10 | <10 |
| Mean T  (August−October) (°C) | 0.21 | 20-25 | 18-20 | 18-15 | 12-15 | <12 |
| Mean T maximum  (April−October) (°C) | 0.08 | 15-24 | 24-26 | 26-28 | 28-30 | <15  >30 |
| Mean T minimum  (April−October) (°C) | 0.08 | 10-15 | 6-10 | 4-6 | 0-4 | <0 |
| Mean T maximum  (August−October) (°C) | 0.24 | 20-30 | 18-20 | 15-18 | 12-15 | >30  <12 |
| **Topography factors** | **0.07** |  |  |  |  |  |
| Slope (%) | 0.07 | 0–12 | 0–12 | 0–12 | 0–12 | >12 |
| **Soil factors** | **0.02** |  |  |  |  |  |
| pH (_CaC12_) (0-30 cm) | 0.02 | 6-7.5 | 5.5-6 | 5.5-5 | 7.5-8 | >8  <5.0 |

**Table S7** Correlation coefficients between detected cold temperature index (*T_C_*) and other important variables used in this analysis. The magnitudes greater than 0.3 are in bold.

| **Variable abbreviation** | **Variable description** | **Units** | **Correlation with Tc** |
| --- | --- | --- | --- |
| Lat | Latitude | Decimal degrees (south) | **0.512** |
| Long | Longitude | Decimal degrees (east) | **-0.308** |
| photoperiod_AO | Average annual day length during April to October | Hours/day | **0.511** |
| PAWC | Plant available water holding capacity | mm | -0.141 |
| GSRain | Average annual growing season rainfall | mm | -0.242 |
| A-ORain | Average annual rainfall during April to October | mm | -0.147 |
| Frost_fsp | Average annual frost days during reproductive and podding period | days | -0.166 |
| MinT_fsp | Average annual minimum daily temperature during the reproductive and podding period | °C | 0.234 |
| meanT_AO | Average annual daily mean temperature during April to October | °C | **0.631** |
| MinT_AO | Average annual daily minimum temperature during April to October | °C | **0.689** |
| Yield | Average annual grain yield | kg/ha | -0.181 |

| **Table S8** Soil chemical and physical properties at the 10 experimental sites. N = nitrate (NO_3_) + ammonium nitrate (NH_4_); OC = organic carbon; LL15 = water content at lower limit of 15-bar; DUL = water content at drained upper limit, and SAT = saturated soil water content | | | | | | | | |
| --- | --- | --- | --- | --- | --- | --- | --- | --- |
| **Site**  **(Latitude**  **Longitude)** | **Soil depth (cm)^$^** | **pH**  **(in 1:5 water)** | **Soil total N (ppm)** | **Soil total OC (%)** | **Bulk density (g/cm^3^)** | **LL15 (mm/mm)** | **DUL (mm/mm)** | **SAT (mm/mm)** |
| Roseworthy  (-34.5106  138.6763) | 0 - 15 | 7.8 | 28.55 | 1.20 | 1.57 | 0.08 | 0.18 | 0.38 |
|  | 15 - 30 | 8.3 | 24.97 | 0.96 | 1.38 | 0.18 | 0.30 | 0.45 |
|  | 30 - 60 | 9.1 | 7.47 | 0.60 | 1.32 | 0.18 | 0.33 | 0.47 |
|  | 60 - 90 | 9.9 | 0.02 | 0.30 | 1.53 | 0.18 | 0.30 | 0.39 |
|  | 90 - 120 | 9.7 | 0.02 | 0.18 | 1.69 | 0.18 | 0.27 | 0.33 |
|  | 120 - 150 | 9.5 | 0.02 | 0.12 | 1.73 | 0.18 | 0.23 | 0.32 |
| Billa Billa  (-28.17  150.4503) | 0 - 15 | 8.2 | 13.99 | 0.95 | 1.36 | 0.20 | 0.40 | 0.46 |
|  | 15 - 30 | 8.8 | 0.91 | 0.95 | 1.37 | 0.20 | 0.40 | 0.45 |
|  | 30 - 60 | 8.9 | 0.89 | 0.96 | 1.39 | 0.22 | 0.39 | 0.45 |
|  | 60 - 90 | 8.0 | 0.80 | 0.85 | 1.43 | 0.22 | 0.38 | 0.43 |
|  | 90 - 120 | 6.0 | 0.02 | 0.55 | 1.46 | 0.22 | 0.37 | 0.42 |
|  | 120 - 150 | 5.1 | 0.02 | 0.26 | 1.46 | 0.22 | 0.37 | 0.42 |
|  | 150 - 180 | 4.7 | 0.02 | 0.20 | 1.42 | 0.22 | 0.38 | 0.43 |
| Roma  (-26.5719  148.7897) | 0 - 10 | 8.2 | 20.98 | 0.92 | 1.29 | 0.20 | 0.40 | 0.48 |
|  | 10 - 20 | 8.5 | 1.40 | 0.78 | 1.32 | 0.22 | 0.41 | 0.46 |
|  | 20 - 40 | 8.6 | 1.39 | 0.35 | 1.32 | 0.24 | 0.42 | 0.47 |
|  | 40 - 60 | 8.6 | 1.27 | 0.23 | 1.33 | 0.24 | 0.42 | 0.47 |
|  | 60 - 70 | 7.4 | 0.01 | 0.15 | 1.39 | 0.24 | 0.40 | 0.45 |
| Wagga Wagga  (-35.0517  147.3493) | 0 - 10 | 6.4 | 14.00 | 1.01 | 1.46 | 0.16 | 0.35 | 0.42 |
|  | 10 - 30 | 6.2 | 8.00 | 0.42 | 1.41 | 0.15 | 0.26 | 0.44 |
|  | 30 - 60 | 6.7 | 5.00 | 0.18 | 1.37 | 0.15 | 0.28 | 0.45 |
|  | 60 - 90 | 6.9 | 2.90 | 0.15 | 1.47 | 0.17 | 0.37 | 0.42 |
|  | 90 - 120 | 6.8 | 3.00 | 0.12 | 1.56 | 0.17 | 0.29 | 0.38 |
| Yenda  (-34.2502  146.1897) | 0 - 15 | 6.0 | 7.88 | 0.59 | 1.68 | 0.14 | 0.26 | 0.34 |
|  | 15 - 30 | 7.1 | 3.36 | 0.37 | 1.52 | 0.14 | 0.30 | 0.40 |
|  | 30 - 60 | 8.1 | 1.30 | 0.05 | 1.42 | 0.15 | 0.35 | 0.43 |
|  | 60 - 90 | 8.0 | 1.25 | 0.05 | 1.52 | 0.19 | 0.31 | 0.40 |
|  | 90 - 120 | 8.1 | 1.26 | 0.07 | 1.56 | 0.19 | 0.33 | 0.38 |
|  | 120 - 150 | 8.3 | 0.02 | 0.07 | 1.46 | 0.19 | 0.33 | 0.42 |
| Trangie  (-31.9861  147.9489) | 0 - 15 | 5.1 | 7.08 | 0.82 | 1.57 | 0.07 | 0.22 | 0.38 |
|  | 15 - 30 | 5.8 | 7.81 | 0.32 | 1.74 | 0.16 | 0.22 | 0.31 |
|  | 30 - 60 | 6.1 | 0.66 | 0.17 | 1.58 | 0.22 | 0.32 | 0.38 |
|  | 60 - 90 | 6.7 | 0.65 | 0.09 | 1.72 | 0.22 | 0.27 | 0.32 |
|  | 90 - 120 | 6.7 | 0.62 | 0.11 | 1.67 | 0.21 | 0.29 | 0.34 |
|  | 120 - 150 | 6.9 | 0.61 | 0.07 | 1.62 | 0.21 | 0.30 | 0.36 |
|  | 150 - 180 | 6.9 | 0.63 | 0.05 | 1.68 | 0.21 | 0.29 | 0.34 |
| Leeton  (-34.5908  146.3679) | 0 - 15 | 7.2 | 21.63 | 1.75 | 1.30 | 0.19 | 0.41 | 0.49 |
|  | 15 - 30 | 7.9 | 3.84 | 1.40 | 1.30 | 0.23 | 0.44 | 0.49 |
|  | 30 - 60 | 8.8 | 1.83 | 0.60 | 1.40 | 0.27 | 0.44 | 0.45 |
|  | 60 - 90 | 9.0 | 1.37 | 0.30 | 1.30 | 0.27 | 0.46 | 0.49 |
|  | 90 - 120 | 8.9 | 1.36 | 0.10 | 1.30 | 0.27 | 0.46 | 0.49 |
|  | 120 - 150 | 8.9 | 0.02 | 0.10 | 1.20 | 0.27 | 0.46 | 0.52 |
|  | 150 - 180 | 8.9 | 0.02 | 0.10 | 1.20 | 0.27 | 0.46 | 0.52 |
| Tamworth  (-31.0867  150.8467) | 0 - 10 | 6.5 | 26.88 | 1.13 | 1.24 | 0.18 | 0.45 | 0.53 |
|  | 10 - 30 | 6.0 | 13.16 | 0.76 | 1.26 | 0.25 | 0.45 | 0.52 |
|  | 30 - 60 | 6.0 | 6.37 | 0.48 | 1.22 | 0.24 | 0.46 | 0.54 |
|  | 60 - 90 | 6.0 | 2.93 | 0.35 | 1.25 | 0.28 | 0.45 | 0.53 |
|  | 90 - 120 | 6.0 | 2.58 | 0.24 | 1.30 | 0.31 | 0.43 | 0.52 |
|  | 120 - 150 | 6.0 | 0.02 | 0.14 | 1.32 | 0.37 | 0.42 | 0.50 |
| Breeza  (-31.1781  150.4240) | 0 - 10 | 8.4 | 12.66 | 1.17 | 1.06 | 0.27 | 0.52 | 0.57 |
|  | 10 - 20 | 8.7 | 11.33 | 0.80 | 1.07 | 0.27 | 0.52 | 0.57 |
|  | 20 - 30 | 8.7 | 10.86 | 0.59 | 1.08 | 0.29 | 0.51 | 0.56 |
|  | 30 - 60 | 8.7 | 2.43 | 0.29 | 1.11 | 0.29 | 0.50 | 0.55 |
|  | 60 - 90 | 8.7 | 1.31 | 0.18 | 1.15 | 0.29 | 0.49 | 0.54 |
|  | 90 - 120 | 8.7 | 1.02 | 0.12 | 1.17 | 0.29 | 0.48 | 0.53 |
|  | 120 - 150 | 8.7 | 0.04 | 0.03 | 1.21 | 0.29 | 0.46 | 0.51 |
| Horsham  (-36.6697  142.1731) | 0 - 10 | 7.7 | 20.99 | 1.30 | 1.26 | 0.17 | 0.45 | 0.50 |
|  | 10 - 25 | 8.5 | 0.64 | 0.50 | 1.21 | 0.12 | 0.47 | 0.52 |
|  | 25 - 40 | 8.9 | 0.74 | 0.65 | 1.39 | 0.18 | 0.40 | 0.45 |
|  | 40 - 55 | 9.1 | 0.66 | 0.33 | 1.37 | 0.18 | 0.41 | 0.46 |
|  | 55 - 70 | 9.1 | 0.63 | 0.20 | 1.37 | 0.18 | 0.41 | 0.46 |
|  | 70 - 100 | 9.1 | 0.56 | 0.19 | 1.26 | 0.18 | 0.44 | 0.49 |
|  | 100 - 130 | 9.1 | 0.02 | 0.13 | 1.26 | 0.18 | 0.44 | 0.49 |

**Supplemental Figures**


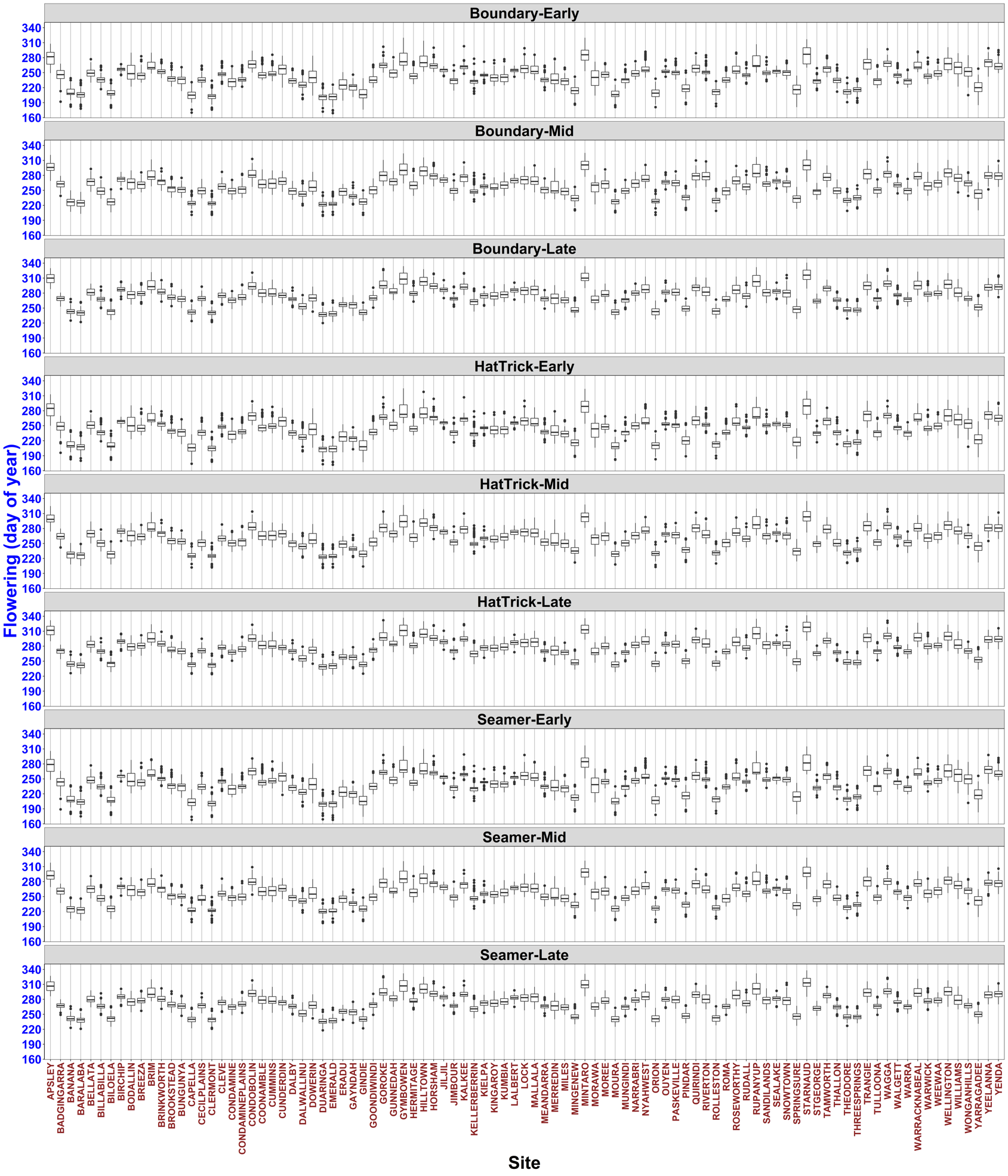


**Fig. S1** Variability of flowering time (1950-2019) across 95 locations (Table S2) under three cultivars (PBA HatTrick, PBA Boundary and PBA Seamer) in three sowing date scenarios (early, mid, and late). The horizontal line in each box-plot is the median value, the lower edge of a box is the 25^th^ percentile and the upper edge, 75^th^. The whiskers reach to 1.5 times the interquartile range (between the 25^th^ and 75^th^ percentiles) or to the most extreme observed value, whichever is smallest; dots below or above the whiskers represent individual values beyond this range.


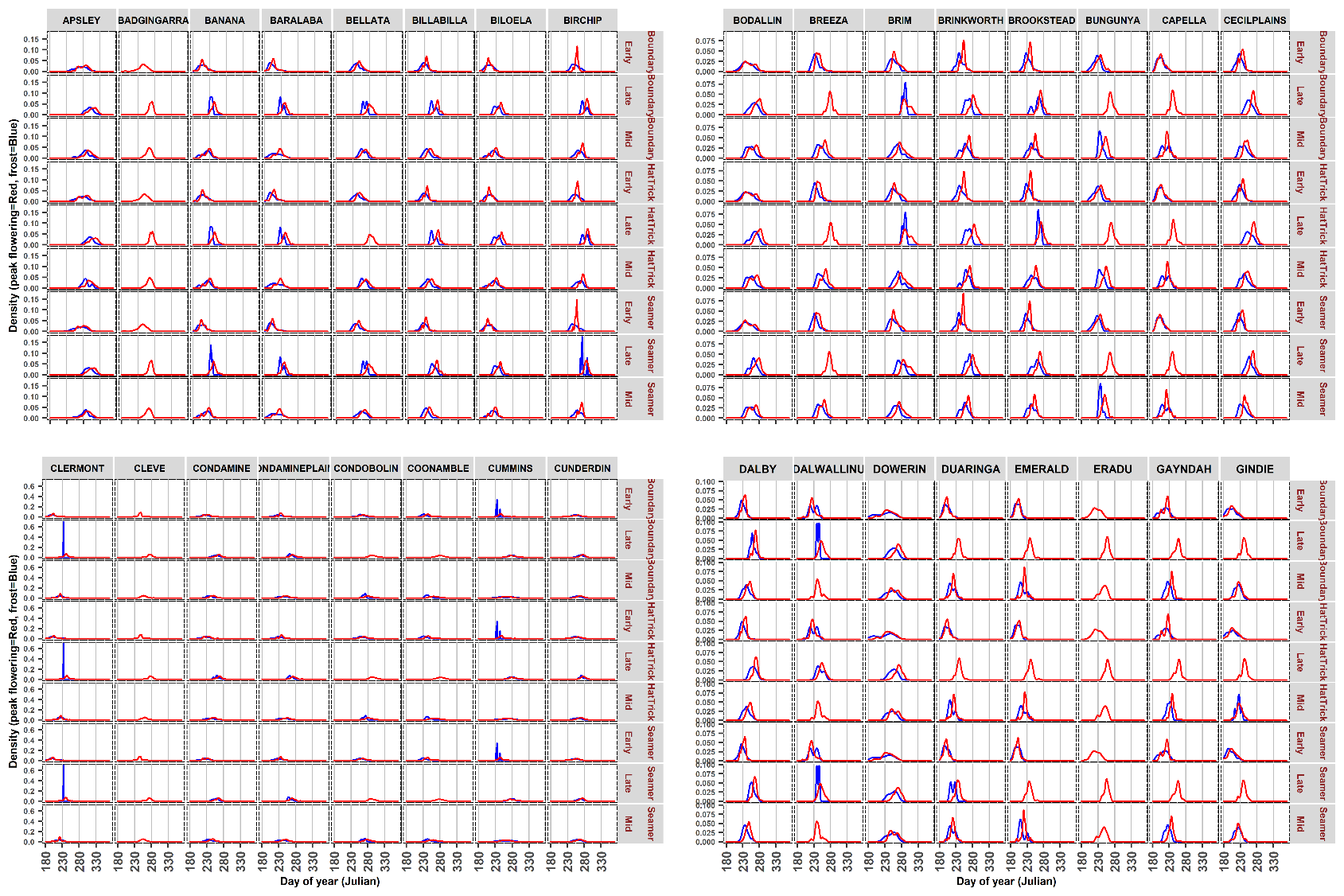


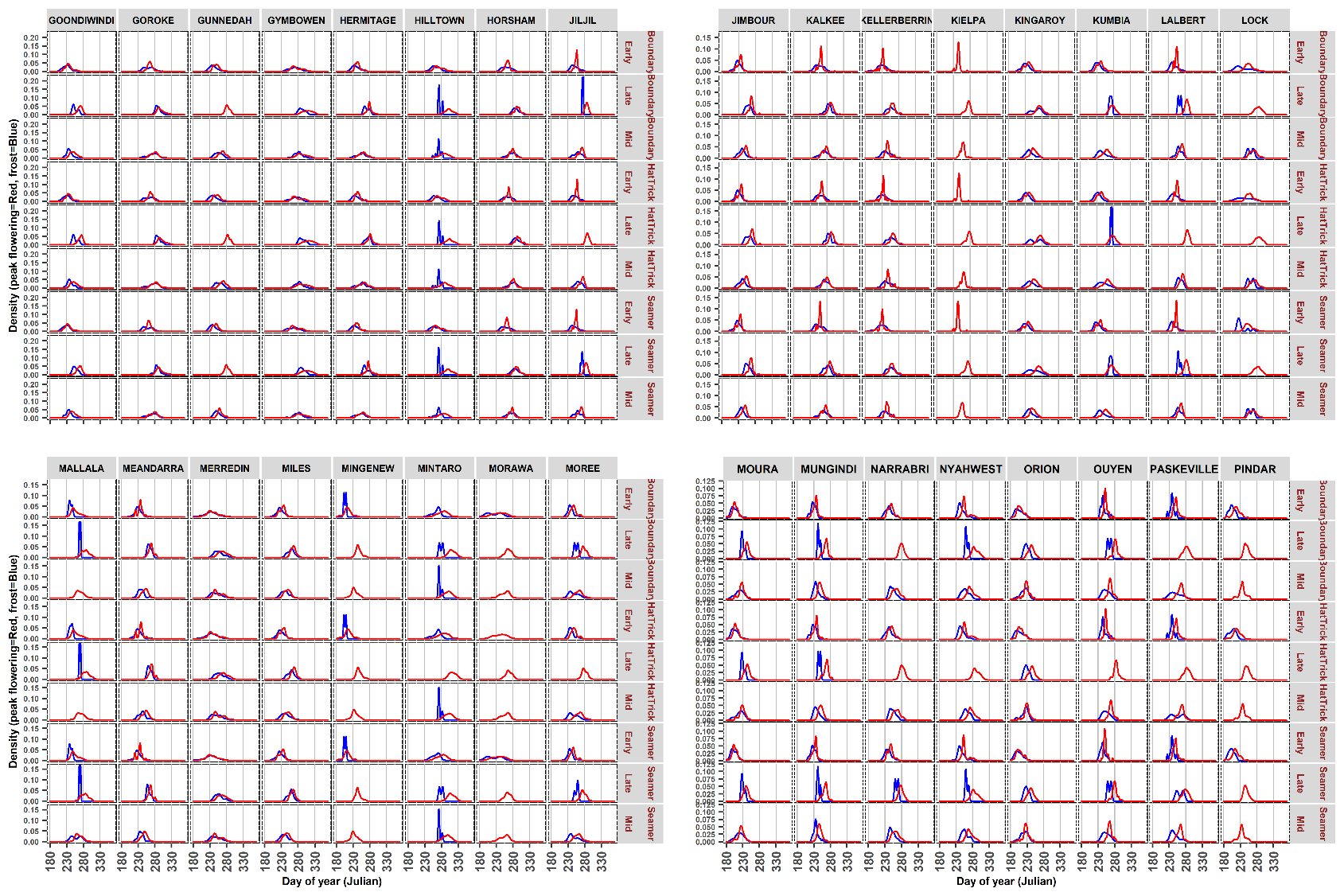


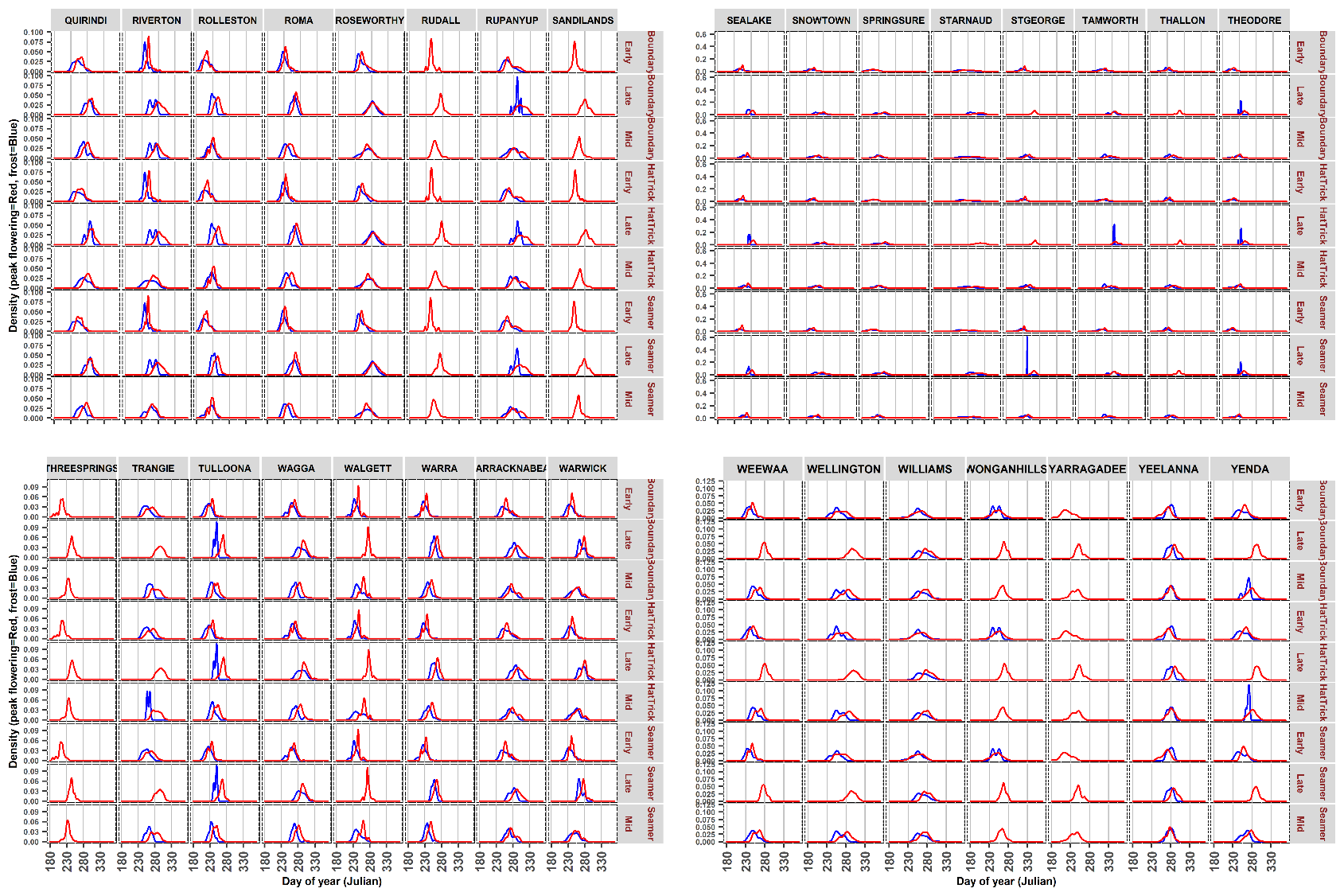


**Fig. S2** The density estimates of frost events (Blue line) and peak flowering time (Red line) for 70 years (1950-2019) across 95 locations. The plots were group by cultivars and sowing dates.


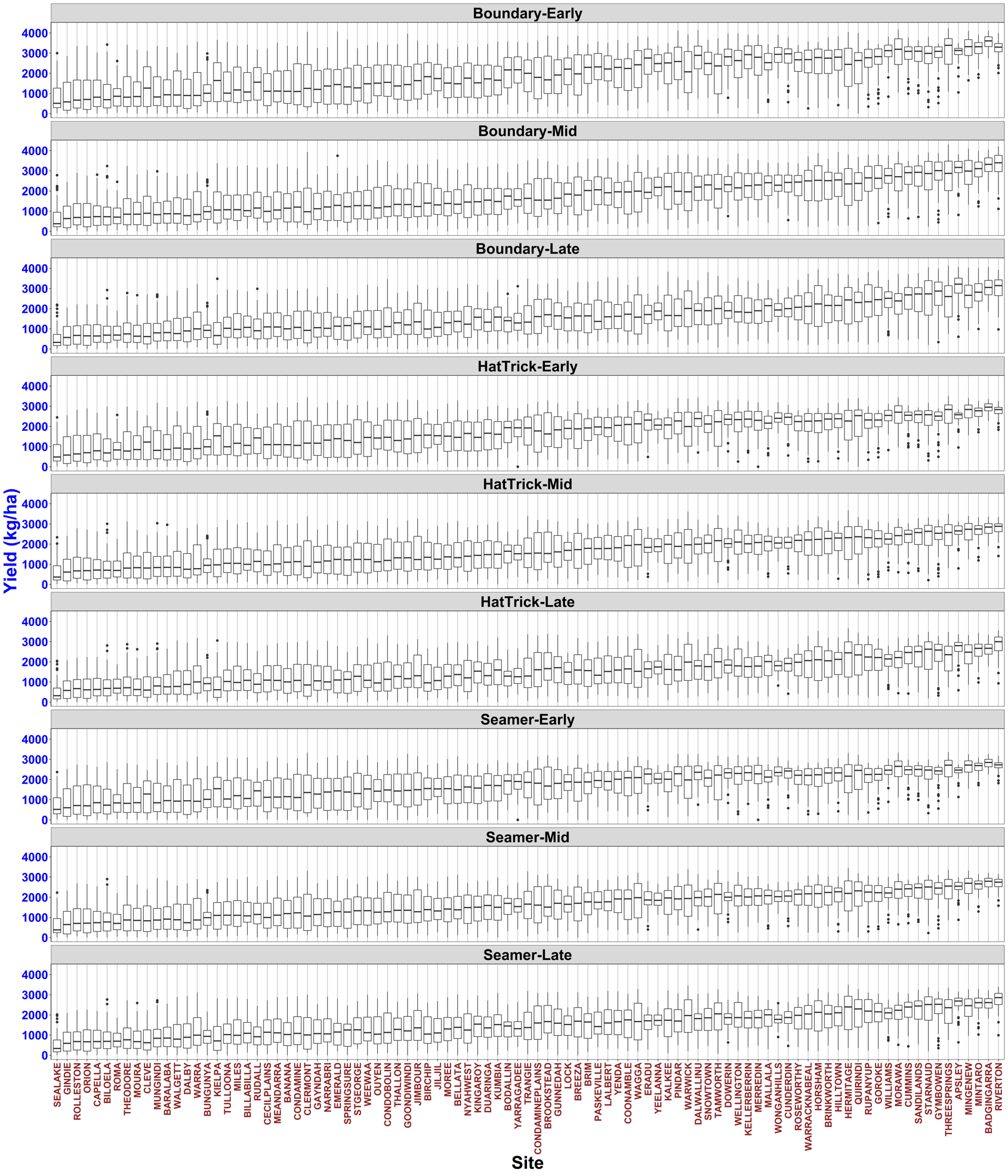


**Fig. S3** Variability of chickpea yields (1950-2019) across 95 locations (Table S3) under three cultivars (PBA HatTrick, PBA Boundary and PBA Seamer) in three sowing-date scenarios (early, mid, and late). The horizontal line in each box-plot is the median value, the lower edge of a box is the 25^th^ percentile and the upper edge, 75^th^. The whiskers reach to 1.5 times the interquartile range (between the 25^th^ and 75^th^ percentiles) or to the most extreme observed value, whichever is smallest; dots below or above the whiskers represent individual values beyond this range.


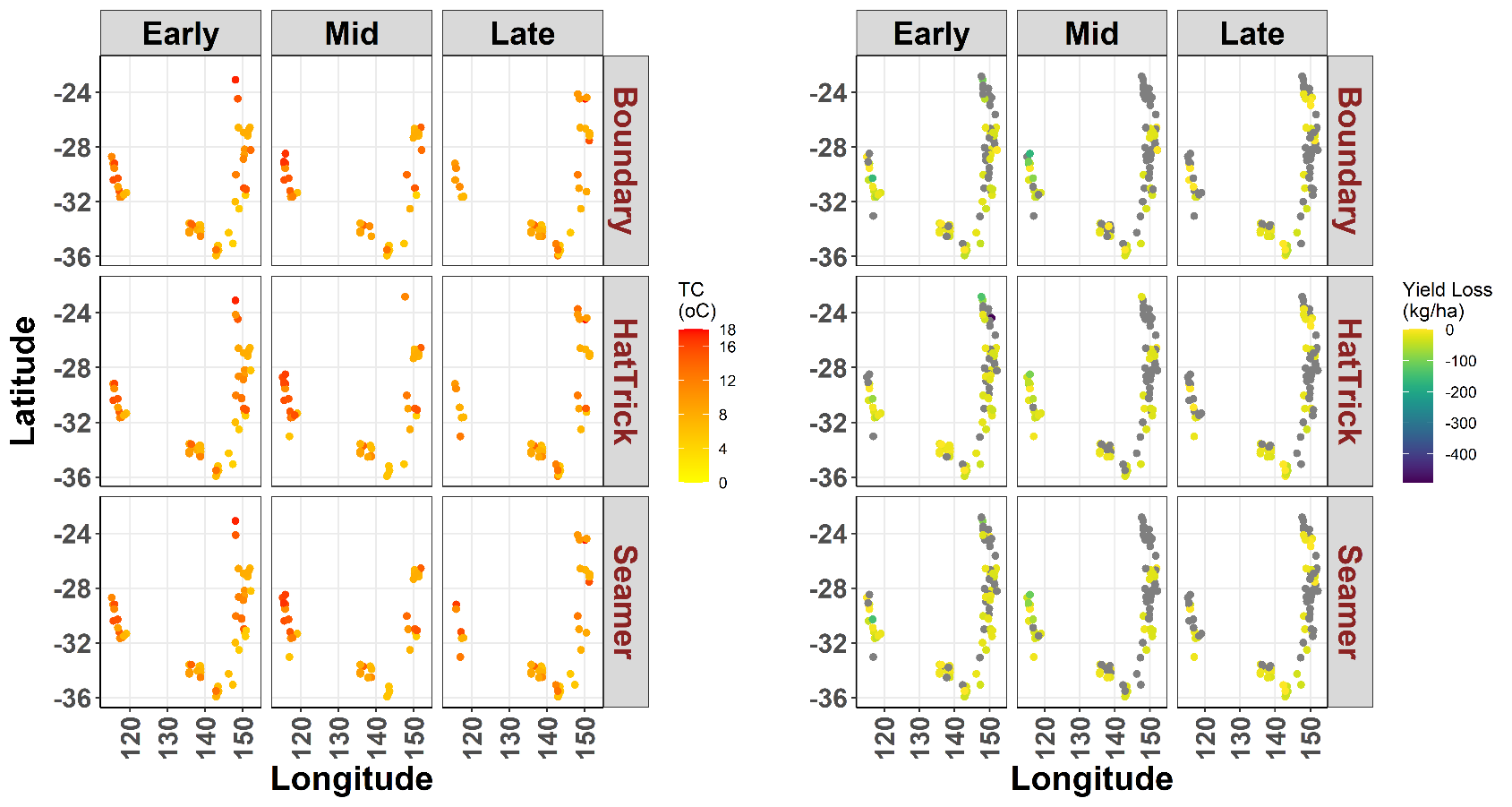


**Fig.** **S4** Cold temperature (*T_C_*) index (left) and the corresponding yield loss (right) against latitude and longitude across 95 locations by cultivar and sowing date scenarios.


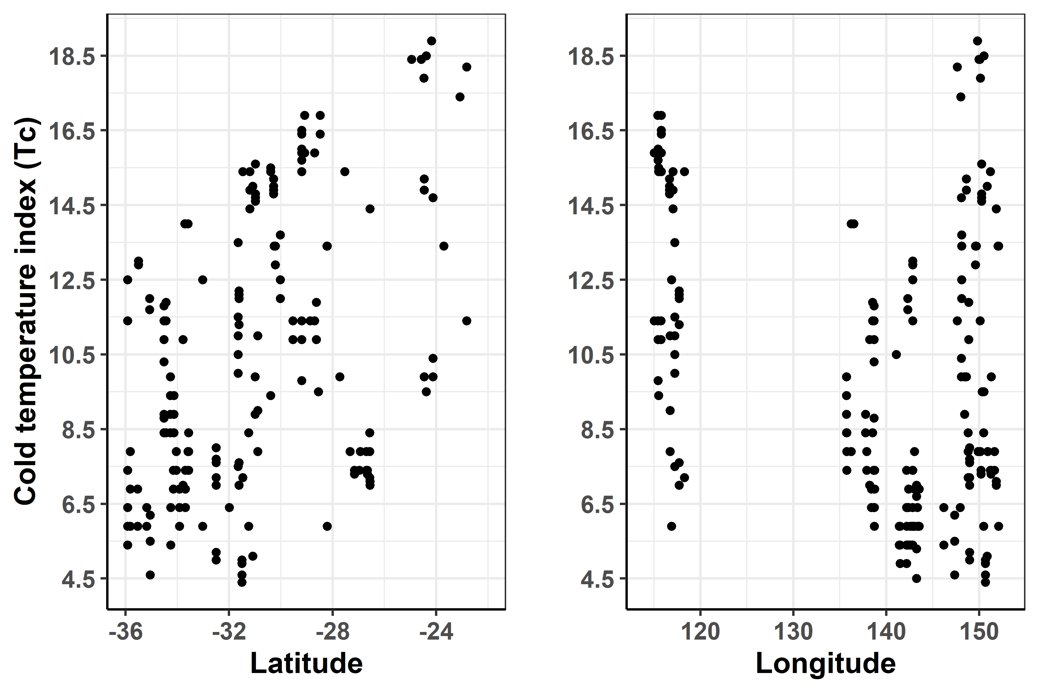


**Fig. S5** Scatterplot of cold temperature (*T_C_*) index versus latitude and longitude across 95 locations where significant value of *T_C_* (p<0.05) was detected (Fig. 6).

**Supplementary References**

Bache SM, Wickham H (2020) magrittr: A Forward-Pipe Operator for R. <https://CRAN.R-project.org/package=magrittr>

Bivand RS, Pebesma E, Gomez-Rubio V (2013) Applied spatial data analysis with R. Springer, New York

Bivand R, Keitt T, Rowlingson B et al (2021) rgdal: Bindings for the 'Geospatial' Data Abstraction Library. <https://CRAN.R-project.org/package=rgdal>

Firke S, Denney B, Haid C et al (2021) janitor: Simple Tools for Examining and Cleaning Dirty Data. <https://CRAN.R-project.org/package=janitor>

Gagolewski M (2021) stringi: Fast and portable character string processing in R. <https://stringi.gagolewski.com>

Garnier S, Ross N, Rudis B et al (2021) viridis: Colorblind-Friendly Color Maps for R. <https://CRAN.R-project.org/package=viridis>

Grolemund G, Wickham H (2011) Dates and Times Made Easy with lubridate. Journal of Statistical Software, 40: 1-25. <https://www.jstatsoft.org/v40/i03/>

Hvitfeldt E (package maintainer) (2021) paletteer: Comprehensive Collection of Color Palettes. <https://CRAN.R-project.org/package=paletteer>

Klik M (2020) fst: Lightning Fast Serialization of Data Frames. <https://CRAN.R-project.org/package=fst>

Bache SM, Wickham H (2020) magrittr: A Forward-Pipe Operator for R. <https://CRAN.R-project.org/package=magrittr>

Müller K (2020) here: A Simpler Way to Find Your Files. <https://CRAN.R-project.org/package=here>

Ooms J (2021) writexl: Export Data Frames to Excel 'xlsx' Format. <https://CRAN.R-project.org/package=writexl>

Pebesma EJ, Bivand RS (2005) Classes and methods for spatial data in R. R News 5. <https://cran.r-project.org/doc/Rnews/>

Pebesma E (2018) Simple Features for R: Standardized Support for Spatial Vector Data. The R Journal, 10: 439-446. <https://doi.org/10.32614/RJ-2018-009>

Pedersen TL (2020). patchwork: The Composer of Plots. <https://CRAN.Rproject.org/package=patchwork>

R Core Team (2021) R: A language and environment for statistical computing. R Foundation for Statistical Computing, Vienna, Austria. <https://www.R-project.org/>

RStudio Team (2021) RStudio: Integrated Development Environment for R. RStudio, PBC, Boston, MA, USA. <http://www.rstudio.com/>

South A (2017) rnaturalearth: World Map Data from Natural Earth. <https://CRAN.R-project.org/package=rnaturalearth>

South A (2017). rnaturalearthdata: World Vector Map Data from Natural Earth Used in 'rnaturalearth'. <https://CRAN.R-project.org/package=rnaturalearthdata>

Thieurmel B, Elmarhraoui A (2019) suncalc: Compute Sun Position, Sunlight Phases, Moon Position and Lunar Phase. <https://CRAN.R-project.org/package=suncalc>

Venables B, Hornik K (2016) oz: Plot the Australian Coastline and States. <https://CRAN.R-project.org/package=oz>

Wickham H (2007) Reshaping Data with the reshape Package. J of Statistical Software 21:1-20. <http://www.jstatsoft.org/v21/i12/>

Wickham H (2016) ggplot2: Elegant Graphics for Data Analysis. Springer-Verlag, New York, USA

Wickham H (2019) stringr: Simple, Consistent Wrappers for Common String Operations. <https://CRAN.R-project.org/package=stringr>

Wickham H, Averick M, Bryan J et al (2019) Welcome to the tidyverse. Journal of Open Source Software, 4: 1686, <https://doi.org/10.21105/joss.01686>

Wickham H, Bryan J (2019) readxl: Read Excel Files. <https://CRAN.R-project.org/package=readxl>

Wickham H (2020) httr: Tools for Working with URLs and HTTP. <https://CRAN.R-project.org/package=httr>

Wickham H (2021) forcats: Tools for Working with Categorical Variables (Factors). <https://CRAN.R-project.org/package=forcats>

Wickham H (2021) tidyr: Tidy Messy Data. <https://CRAN.R-project.org/package=tidyr>

Wickham H, Hester J (2020) readr: Read Rectangular Text Data. <https://CRAN.R-project.org/package=readr>
